# Global distribution models for the major bamboo (Poaceae, Bambusoideae) clades

**DOI:** 10.3897/BDJ.13.e153436

**Published:** 2025-08-26

**Authors:** Christopher Tyrrell

**Affiliations:** 1 Milwaukee Public Museum, Milwaukee, United States of America Milwaukee Public Museum Milwaukee United States of America

**Keywords:** Bambusoideae, distribution models, maps, MaxEnt

## Abstract

**Background:**

The bamboos (Poaceae, Bambusoideae) are important ecological and economic resources distributed across five continents. Maps of the distribution of the four major bamboo clades are popular in the scientific and trade literature. To date, these global scale maps have been drawn manually through various means. Despite their general accuracy, previous maps lack the ability to repeatably reconstruct them as new data becomes available.

**New information:**

This work develops a workflow and predicts potential and realised distribution maps for the four major bamboo clades using MaxEnt "species" distribution modelling, based on vetted natural history specimen records. The source data, scripts and resultant products are provided creating a Findable, Accessible, Interoperable and Reusable (FAIR) dataset and workflow.

## Introduction

The bamboo subfamily (Poaceae, Bambusoideae) contains over 1,700 described species distributed across five continents (Ohrnberger 1999). Molecular phylogenetic inference has repeatedly revealed four major clades within Bambusoideae: temperate woody bamboos (TWB), palaeotropical woody bamboos (PWB), neotropical woody bamboos (NWB) and herbaceous bamboos (Clark et al. 1995, Grass Phylogeny Working Group 2001, Kelchner and Bamboo Phylogeny Group 2013). The herbaceous and temperate woody bamboo clades each correspond to a formal nomenclatural rank (tribes Olyreae and Arundinarieae, respectively), while the tropical woody clades are informal groups that subdivide tribe Bambuseae into species native either east (palaeo-) or west (neo-) of the Atlantic Ocean. This high-level biogeographic partitioning has made maps of the distribution of bamboo clades popular in the scientific and trade literature (e.g. Ramakrishnan et al. (2020), Adeniyi et al. (2022), Suganthasakthivel (2023), Ma et al. (2024), Ruiz-Sanchez (2025)).

Most of the maps are either hand drawn or derivatives of hand-drawn maps. While generally accurate, these hand-drawn maps lack a rigorous basis.

My objectives are two fold: 1) to develop a reproducible workflow for and produce GIS files of the potential distributions of the four major bamboo clades using species distribution modelling (e.g. Elith and Leathwick (2009)), based on vetted natural history specimen records and 2) to produce map images of the estimated, realised distributions of each clade and combinations of clades from two different hemispherical perspectives. I provide the inputs, scripts and resultant products as both geospatial data and image files under an Open Data Commons licence.

## Sampling methods

### Sampling description

All physical specimen records of Poaceae with geocoordinates were downloaded from GBIF on 26 Nov 2024 (GBIF 2024; 1,793,141 records). The records were then filtered to include only bamboo genera (Table 1; 44,513 records remaining). The dataset was truncated to the following eight columns: gbifID, datasetKey, genus, decimalLatitude, decimalLongitude, coordinateUncertaintyInMeters, stateProvince and countryCode. Data refinement and modelling were conducted in R v.4.4.1 (R Core Team 2024) with RStudio v.2024.12.0+467 (Posit team 2024), scripts used here being available in Suppl. material 1.

### Quality control

Records were next filtered to include only those that occur within countries or states/provinces where a genus is considered native according to Vorontsova et al. (2016) and the protologues for more recent genera (Tyrrell et al. 2018, Zhang et al. 2018, Haevermans et al. 2020, Qin et al. 2020, Tong et al. 2020, Zhang et al. 2020, Jesus-Costa et al. 2024 and Oliveira et al. 2024). This white list approach was done to eliminate putative cultivated specimens and was performed in R by looping over the genera and matching the country code in Suppl. material 2 with the applicable records from GBIF, including only those for which the GBIF country codes or, in the case of Australia, China, Russia and the United States, the country code + state/province name, matched.

The remaining records were further filtered for geocoordinate quality using the CoordinateCleaner package (Zizka et al. 2019). The two-letter country codes from GBIF were converted to three-letter codes by matching against ISO (2020) for compatibility with CoordinateCleaner. Coordinates were removed if they were invalid (cc_val; 0 records removed), exact duplicates within a genus (cc_dupl; 13,889 records matched), beyond 0.5 km from a coastline (cc_sea; 2,775 records matched), were within 999 m of the centroid for a country or first-level administrative division (cc_cen; 1,369 records matched), were outside of the country indicated (cc_coun; 32,813 records matched) or their mean distance from other points of the same genus exceeded 10 times the interquartile range of the other points for that genus (cc_outl; 190 records matched). The final, filtered points and attribute table are available in the Suppl. material 3 geopackage file.

### Step description

Distribution modelling used the 19 bioclimatic variables of Fick and Hijmans (2017) and the cleaned bamboo specimen points (Suppl. material 3). Maximum Entropy (MaxEnt) modelling (Phillips et al. 2006) was used to predict clade distributions. The MaxEnt algorithm adjusts for redundant variables and is, therefore, relatively robust to the multicollinearity among the bioclimatic variables (Feng et al. 2019); therefore, no dimension-reduction procedure was conducted. For the herbaceous and neotropical woody clades, the specimen points were randomly partitioned into training (67%) and test (33%) sets. Palaeotropical woody points were partitioned in an 80% training, 20% testing split because there were fewer non-redundant data points. The temperate woody bamboos were partitioned into two parts for modelling: points in Asia and North America (67:33 training/test) vs. points in Africa (80:20 training/test). This was done due to the extreme imbalance of specimens records among the continents (6,488 vs. 58, respectively). The high number of northerly points completely overwhelmed any signal from southern latitudes and the unsplit model failed to predict any of the known regions on the African continent. MaxEnt distribution modelling was performed using the maxent() function of the dismo package v.1.3-16 (Hijmans et al. 2010). Model performance was evaluated using the target clade test set points and background points drawn at random from each of the non-target clades (exclusive of overlapping points). The models were then used to predict clade probability back on to the bioclim raster layers. The partitioned TWB model predictions were combined into a raster stack [raster::stack()] and merged by taking the maximum cell value between the two raster layers [calc(fun = max)].

The predicted, potential distributions were exported as geoTIFFs with world files (Suppl. material 4) using the writeRaster() function (raster package v.3.6-30; Hijmans (2010)) in two different sets. The first set has a single, continuous band representing the probability of occurrence [0-1]. The second set has a single band representing a boolean value [0, 1] indicating whether the probability of occurrence for the clade exceeded the threshold value of the maximum summed sensitivity and specificity (“spec_sens”). In the TWB, where the data were partitioned by continent and later merged, the threshhold value from the larger, northern data model was used to discretise the merged output.

Map image files (Suppl. material 5) were generated using QGIS v.3.24 (QGIS 2022). The world boundary baselayer of the maps was derived from GADM v.4.1 (GADM 2022). Binary (threshold) raster files were imported as well as vector specimen data points. The predicted clade distribution was expected to encompass regions where no input data points were present (potential distribution). These regions represent areas where habitat may be suitable for members of the clade, but where, for a variety of reasons, no records of those organisms occur (Townsend Peterson et al. 2011). As my second objective was to produce maps of the known (realised) distributions for each clade, I performed a data-masking step whereby the full model predictions were reduced using a mask layer (sensu Merow et al. (2022)). The mask layer was generated by creating circular buffers around the cleaned and filtered data points for each clade. The overlapping borders among these circles were dissolved to create contiguous polygons (see Suppl. material 4). The influence of each point is assumed to decay with distance (Cressie 2015). I therefore chose to use 25% of the median inter-point distance as the diameter for the buffered circles. This was determined by first calculating the median distance between all points of each clade, then the median of these four clade values was divided by 4 (resulting in a value ~ 6.35 decimal degrees). The buffers were generated in QGIS using this radius and six segments to estimate a quarter circle. Raster predictions were extracted using QGIS's 'Clip Raster by Mask Layer' extraction function.

Map images are provided in two alternative projections: Atlantic-centric, EPSG 8857 centred at coordinates 0° N, 0° E; and Pacific-centric, EPSG 8859 centred at 0° N, 150° E (e.g. Fig. 1).

## Geographic coverage

### Description

The maps and geopackages are worldwide in extent (90° North and South Latitude, 180° East and West Longitude). The resultant rasters have a graticule-based grain size of 5 arc-minutes (~ 0.083°); therefore; raster cells represent progressively smaller areas from the Equator toward the Poles (approximately 86 km^2^ at the Equator, 72 km^2^ at 23.44° and 43 km^2^ at 45° latitude).

## Taxonomic coverage

### Taxa included

**Table:** 

Rank	Scientific Name	
subfamily	Bambusoideae	

## Temporal coverage

**Data range:** 1817-1-01 – 2024-11-26.

## Usage licence

### Usage licence

Open Data Commons Attribution License

### IP rights notes

These model outputs are made available under the Open Data Commons Attribution Licence: http://opendatacommons.org/licenses/by/1.0/

## Data resources

### Data package title

Preserved specimen occurrences for Poaceae GBIF

### Resource link

https://doi.org/10.15468/dl.u9wq7c

### Alternative identifiers

https://www.gbif.org/occurrence/search?basis_of_record=PRESERVED_SPECIMEN&issue=GEODETIC_DATUM_ASSUMED_WGS84&issue=CONTINENT_DERIVED_FROM_COORDINATES&taxon_key=3073

## Additional information

### Discussion

Evaluation of model performance was based on area under the receiver operating characteristic curve (AUC). AUC values can range from 100% (perfectly ranks positive cases higher than negative) to 50% (no better than random). Overall, all models seemed to perform moderately well. The PWB had the weakest performing model (AUC ~ 85.1%) and the TWB the strongest (AUC ~ 98.3% [northern partition], AUC ~ 95.2% [Africa partition]), with herbaceous (AUC ~ 86%) and NWB (AUC ~ 92.3%) falling between. Model correlations mirror the AUC assessments: TWB [northern partition] = 0.874, TWB [Africa partition] = 0.722, NWB = 0.570, herbaceous 0.404, PWB = 0.185.

Comparison of the masked outputs (Suppl. material 5) with prior published maps reveal both interesting nuances and spurious artefacts. The maps produced here are fairly comparable to the most frequently used maps (Clark et al. 2005) and a more recently created map (fig. 1a) from Ma et al. (2024).

Among the Olyreae (herbaceous bamboos), the most notable difference from Clark et al. (2005) is the exclusion of this tribe from Africa. This was an intentional result of the specimen filtering process. Soderstrom and Zuloaga (1989) suggest *Olyra* was introduced to Africa and Ruiz-Sanchez et al. (2019) provided population genetic evidence suggestive of a recent, potentially anthropogenic, introduction of the genus to Africa. Therefore, all data points for this clade were excluded from Africa. Despite this, the potential (unmasked) distributions generated here (Suppl. material 4) do identify central Africa as candidate habitat for the herbaceous clade. The point-buffer masking process eliminated these areas from the images (Suppl. material 5).

The herbaceous map produced here also predicts unsuitable habitat in central and east-central Brazil, which does not appear in either Clark et al. (2005) or Ma et al. (2024). This area roughly corresponds to the Cerrado ecoregion, drier savannah-like habitat for which there are few herbaceous bamboo specimens. The pattern is echoed in the drier western parts of Chile and Peru where the MaxEnt models do not predict a high probability of Olyreae, but both previous maps show the clade reaching the coast. One interesting artefact is the potential herbaceous habitat in south-central Florida. Soderstrom and Zuloaga (1989) mention seeing a photograph of a herbarium sheet from the Chapman Herbarium comprising a mixed collection of *O. latifolia* from Florida.

The realised distribution for the NWB clade mainly differs from the published maps in the glaring absence of habitat that exceeds threshold levels in the Amazon Basin. Despite specimen records from this area, their density appears to be too sparse for the model to predict a high probability of occurrence. This is similar to the challenge I experienced with the unsplit TWB model not predicting the TWB clade to occur in Africa and is a limitation of the distribution modelling process. Interestingly, however, the model does predict the NWB clade in Guianan savannahs of north-eastern South America. This is an area where some near-endemic taxa (e.g. *Myriocladus*) are known to occur and, perhaps because of this, has a higher density of collections. The narrow distribution of NWB in the southern Andes shown in Clark et al. (2005) and Ma et al. (2024) is also detected in this study. However, the MaxEnt threshold predictions produce a discontinuity in the NWB range beginning in northern Argentina. The distribution for NWB resumes further south, but appears to be predicted down into the Magallanes Region of southern Chile, which may be influenced by the single data point around 46.72° South latitude.

Modelling the distribution of the PWB was challenged by the fewer specimen records (1,202) for this clade relative to the others (herbaceous = 4,274, NWB = 5,586, TWB = 7,586). Considering this clade ranges across Africa, Asia and into northern Australia (minimally 15,700 km longitudinally), the possible data density (0.077 points/km) is nearly an order of magnitude lower than that for the NWB (0.570 points/km), which only range from southern North America into southern South America (approximately 9,800 km latitudinally). I believe this accounts for some of the contrasts observed between the maps produced here and those previously published. In particular, the predicted occurrence of PWB in Africa and India is much smaller and discontinuous when compared with Clark et al. (2005) and Ma et al. (2024). Not all deviations from prior maps, however, are necessarily failures of the models. In Australia, for example, the Clark et al. (2005) and Ma et al. (2024) maps generically highlight most of the Cape York Peninsula, but the MaxEnt maps more realistically identify the coastal rainforest areas from which specimens are known and exclude the savannah and shrublands of the interior. Similar patterns appear in Madagascar, Borneo and the windward slopes of the Himalayas.

As described in the methods, initial modelling of the TWB failed to predict any occurrence (even in the continuous output) in Africa. Thus, I elected to partition the data into the 58 points from the African continent and the 7,528 from Asia and North America, model these independently, then merge the results by taking the maximum predicted value of each grid cell from either of the two model outputs. This achieved the desired effect —predicted distributions in Africa— at the cost of being able to directly compare the TWB models to those of the other clades. In broad strokes, the TWB maps here and those from the literature paint equivalent pictures of the realised TWB distribution. The main omissive deviations include a lack of predicted range into mainland Southeast Asia and the Malay Peninsula (depicted in both the published maps) and the absence of TWB from Madagascar. The latter result occurs despite the availability of TWB specimens from the island. The chief additive deviation is the appearance of a range artefact in eastern Mexico (where no collections of TWB have ever been made).

Maps are models and all models are simplifications (Kokko 2005). The products provided here are no exception. Each of the clade maps I generated and provided have both information and error associated with them and their use or application should always be considered on a case-by-case basis. With that caveat, I proffer the pre-made images (Suppl. material 5) as statistically estimated, specimen-based predictions of the putatively native distributions for each of the four major bamboos clades. I have provided my entire workflow (Suppl. materials 1, 3) and data products (Suppl. material 4) here so they are Findable, Accessible, Interoperable and Reusable. I encourage others to use these to improve upon or generate new maps for one of the most remarkable subfamilies of grass.

## Supplementary Material

D4098F76-4AA3-5AED-8837-0C4E751DFAC9 10.3897/BDJ.13.e153436.suppl1 Supplementary material 1 R script to clean occurrence records and model distributions Data type text/r-script Brief description Script in R-language to clean occurrence records and model distributions. File: oo_1357084.R https://binary.pensoft.net/file/1357084 Christopher D. Tyrrell

0EFB826E-9796-553F-88F3-52799599A8C3 10.3897/BDJ.13.e153436.suppl2 Supplementary material 2 Native regions for bamboo genera based on literature sources Data type text/csv Brief description Regions to which bamboo genera are considered native and, thus, included in distribution modelling. Region column contains the 2-letter country code or 2-letter country code and name of a state or province separated by a colon. Table is compiled from published sources: Vorontsova et al. (2016), Tyrrell et al. (2018), Zhang et al. (2018), Haevermans et al. (2020), Qin et al. (2020), Tong et al. (2020), , Zhang et al. (2020), Jesus-Costa et al. (2024) and Oliveira et al. (2024). File: oo_1394389.csv https://binary.pensoft.net/file/1394389 Christopher D. Tyrrell

3C588DEC-7CE8-507F-8945-4D76A04315F7 10.3897/BDJ.13.e153436.suppl3 Supplementary material 3 Bambusoideae points csv Data type text/csv (occurrences) Brief description Geopackage containing vector point layer of Bambusoideae (Poaceae) occurences filtered from GBIF (https://doi.org/10.15468/dl.u9wq7c). File: oo_1357085.csv https://binary.pensoft.net/file/1357085 Christopher D. Tyrrell

67067A0B-6D8E-50A8-BB1E-FC182953E370 10.3897/BDJ.13.e153436.suppl4 Supplementary material 4 Model output (GIS input) files of estimated distributions of Bambusoideae clades Data type zip file containing image/geotiff, application/geopackage+sqlite3 Brief description Directory containing model output files (geoTIFF and World files, or geopackages) of estimated distributions for Bambusoideae clades. Subdirectory 'continuous' contains geoTIFFs with raster cells assigned MaxEnt probabilities 0 to 1; subdirectory 'binary' contains geoTIFFs with raster cells coded 0 (less than or equals threshold probability) or 1 (exceeds threshold probability) as determined by maximum summed sensitivity and specificity. Subdirectory 'binary' is further subdivided into 'raster - raw' (geoTIFFs of the unaltered model predictions), 'raster - masked' (geoTIFFs of the model predictions masked using the buffers around points) and 'polygonised' (geopackages of the raster - masked files that have been converted to vector format). Subdirectory 'mask' contains geopackages with the 6.35 decimal degree buffers around data points that were used to mask (exclude) distribution predictions. Subdirectory 'points' contains geopackages of the final data points for each clade.
nwb = neotropical woody; pwb = palaeotropical woody; twb = temperate woody; herb = herbaceous (Olyreae); woody = nwb + pwb + twb File: oo_1357086.zip https://binary.pensoft.net/file/1357086 Christopher D. Tyrrell

3CF10775-756B-5DC8-9406-AD78D6661DA3 10.3897/BDJ.13.e153436.suppl5 Supplementary material 5 Map images (png) of estimated Bambusoideae clade distributions Data type zip file containing image/png (maps) Brief description Directory containing png maps of estimated Bambusoideae clade distributions produced with a GIS, in two alternate projections: Atlantic Ocean centred (EPSG 8857) or Pacific Ocean centred (EPSG 8859). Further subdirectories offer alternative display of information: areas, points or points+areas (points overlaid on coloured areas).
nwb = neotropical woody bamboos; pwb = palaeotropical woody bamboos; twb = temperate woody bamboos; woody = nwb + pwb + twb; all = woody + herbaceous File: oo_1357087.zip https://binary.pensoft.net/file/1357087 Christopher D. Tyrrell

## Figures and Tables

**Figure 1a.**
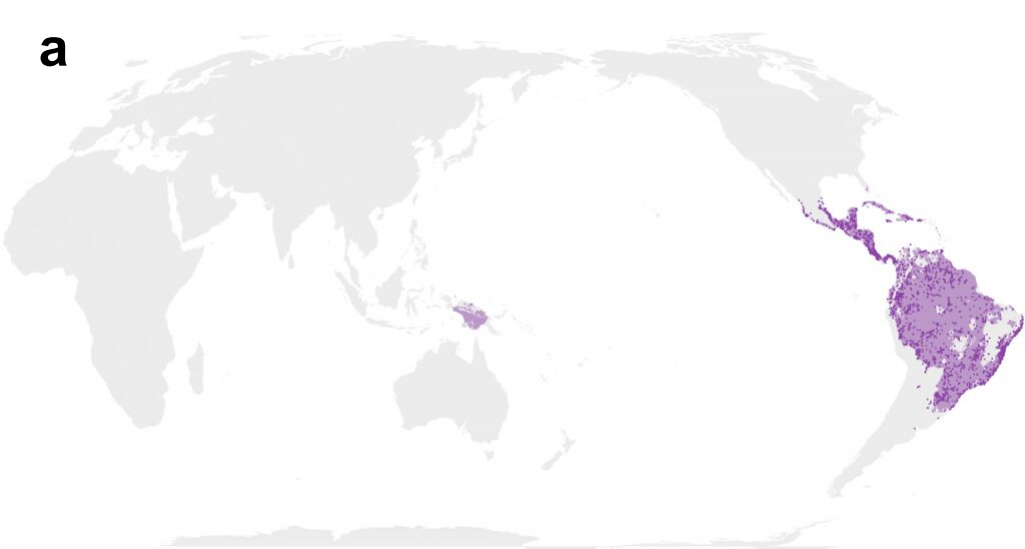


**Figure 1b.**
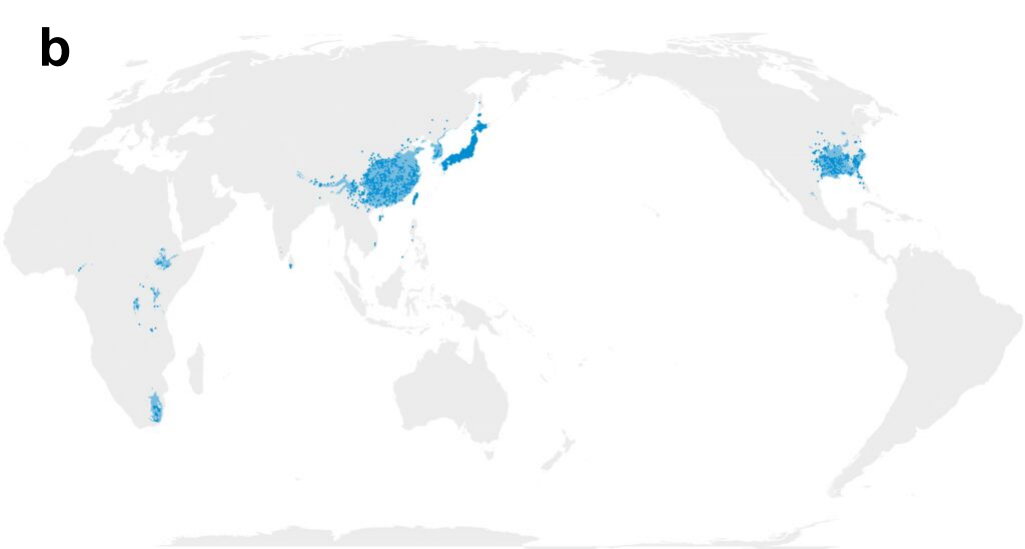


**Figure 1c.**
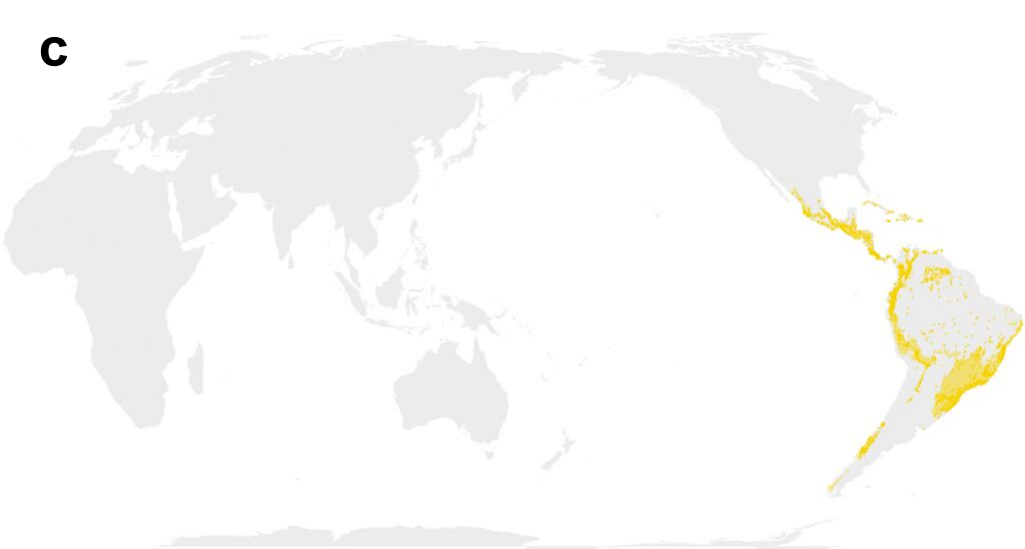


**Figure 1d.**
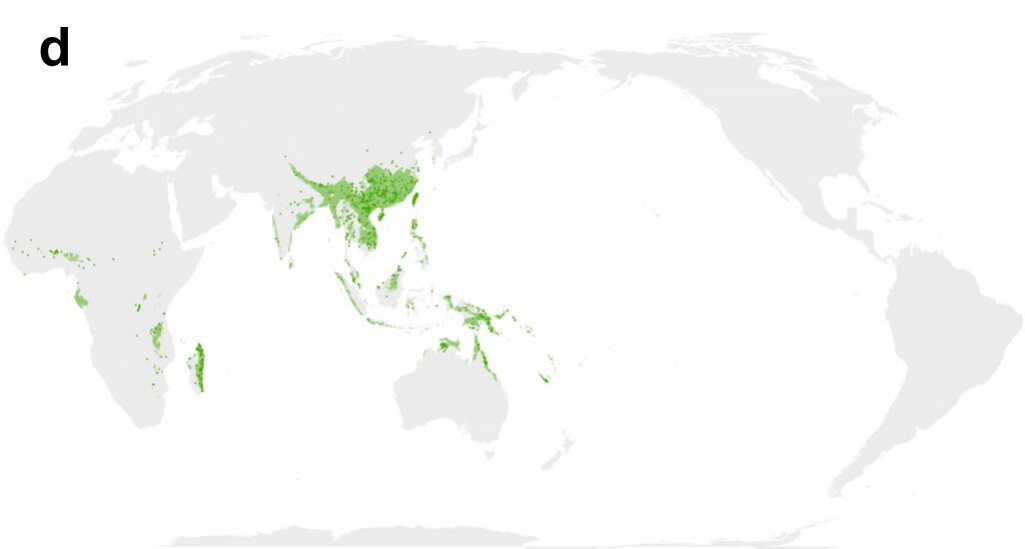


**Table 1.** Tribe, subtribe and clade classifications for bamboo genera used here. Clade identifies the informal clade name: NWB = neotropical woody bamboos, PWB = palaeotropical woody bamboos, TWB = temperate woody bamboos. Classification generally follows Kelchner and Bamboo Phylogeny Group (2013), Zhang et al. (2020) and Liu et al. (2024).

Tribe	Informal Clade	Subtribe	Genus
Olyreae	Herbaceous	Buergersiochloinae	* Buergersiochloa *
Olyreae	Herbaceous	Buergersiochloinae	* Ekmanochloa *
Olyreae	Herbaceous	Buergersiochloinae	* Mniochloa *
Olyreae	Herbaceous	Buergersiochloinae	* Piresiella *
Olyreae	Herbaceous	Olyrinae	* Agnesia *
Olyreae	Herbaceous	Olyrinae	* Arberella *
Olyreae	Herbaceous	Olyrinae	* Brasilochloa *
Olyreae	Herbaceous	Olyrinae	* Cryptochloa *
Olyreae	Herbaceous	Olyrinae	* Diandrolyra *
Olyreae	Herbaceous	Olyrinae	* Froesiochloa *
Olyreae	Herbaceous	Olyrinae	* Lithachne *
Olyreae	Herbaceous	Olyrinae	* Maclurolyra *
Olyreae	Herbaceous	Olyrinae	* Olyra *
Olyreae	Herbaceous	Olyrinae	* Parodiolyra *
Olyreae	Herbaceous	Olyrinae	* Piresia *
Olyreae	Herbaceous	Olyrinae	* Raddia *
Olyreae	Herbaceous	Olyrinae	* Raddiella *
Olyreae	Herbaceous	Olyrinae	* Rehia *
Olyreae	Herbaceous	Olyrinae	* Reitzia *
Olyreae	Herbaceous	Olyrinae	* Sucrea *
Olyreae	Herbaceous	Olyrinae	* Taquara *
Olyreae	Herbaceous	Parianinae	* Aemulanthus *
Olyreae	Herbaceous	Parianinae	* Eremitis *
Olyreae	Herbaceous	Parianinae	* Pariana *
Olyreae	Herbaceous	Parianinae	* Parianella *
Bambuseae	NWB	Arthrostylidiinae	* Actinocladum *
Bambuseae	NWB	Arthrostylidiinae	* Alvimia *
Bambuseae	NWB	Arthrostylidiinae	* Arthrostylidium *
Bambuseae	NWB	Arthrostylidiinae	* Athroostachys *
Bambuseae	NWB	Arthrostylidiinae	* Atractantha *
Bambuseae	NWB	Arthrostylidiinae	* Aulonemia *
Bambuseae	NWB	Arthrostylidiinae	* Cambajuva *
Bambuseae	NWB	Arthrostylidiinae	* Colanthelia *
Bambuseae	NWB	Arthrostylidiinae	* Didymogonyx *
Bambuseae	NWB	Arthrostylidiinae	* Elytrostachys *
Bambuseae	NWB	Arthrostylidiinae	* Filgueirasia *
Bambuseae	NWB	Arthrostylidiinae	* Glaziophyton *
Bambuseae	NWB	Arthrostylidiinae	* Merostachys *
Bambuseae	NWB	Arthrostylidiinae	* Myriocladus *
Bambuseae	NWB	Arthrostylidiinae	* Quixiume *
Bambuseae	NWB	Arthrostylidiinae	* Rhipidocladum *
Bambuseae	NWB	Arthrostylidiinae	* Stelanemia *
Bambuseae	NWB	Arthrostylidiinae	* Vianaea *
Bambuseae	NWB	Chusqueinae	* Chusquea *
Bambuseae	NWB	Guaduinae	* Apoclada *
Bambuseae	NWB	Guaduinae	* Eremocaulon *
Bambuseae	NWB	Guaduinae	* Guadua *
Bambuseae	NWB	Guaduinae	* Olmeca *
Bambuseae	NWB	Guaduinae	* Otatea *
Bambuseae	NWB	Guaduinae	* Tibisia *
Bambuseae	PWB	(incertae sedis)	* Ruhooglandia *
Bambuseae	PWB	Bambusinae	* Bambusa *
Bambuseae	PWB	Bambusinae	* Bonia *
Bambuseae	PWB	Bambusinae	* Cochinchinochloa *
Bambuseae	PWB	Bambusinae	* Dendrocalamus *
Bambuseae	PWB	Bambusinae	* Gigantochloa *
Bambuseae	PWB	Bambusinae	* Laobambos *
Bambuseae	PWB	Bambusinae	* Maclurochloa *
Bambuseae	PWB	Bambusinae	* Melocalamus *
Bambuseae	PWB	Bambusinae	* Neomicrocalamus *
Bambuseae	PWB	Bambusinae	* Oreobambos *
Bambuseae	PWB	Bambusinae	* Oxytenanthera *
Bambuseae	PWB	Bambusinae	* Phuphanochloa *
Bambuseae	PWB	Bambusinae	* Pseudoxytenanthera *
Bambuseae	PWB	Bambusinae	* Soejatmia *
Bambuseae	PWB	Bambusinae	* Temochloa *
Bambuseae	PWB	Bambusinae	* Thyrsostachys *
Bambuseae	PWB	Bambusinae	* Vietnamosasa *
Bambuseae	PWB	Bambusinae	* Yersinochloa *
Bambuseae	PWB	Dinochloinae	* Cyrtochloa *
Bambuseae	PWB	Dinochloinae	* Dinochloa *
Bambuseae	PWB	Dinochloinae	* Mullerochloa *
Bambuseae	PWB	Dinochloinae	* Neololeba *
Bambuseae	PWB	Dinochloinae	* Parabambusa *
Bambuseae	PWB	Dinochloinae	* Pinga *
Bambuseae	PWB	Dinochloinae	* Sphaerobambos *
Bambuseae	PWB	Greslaniinae	* Greslania *
Bambuseae	PWB	Hickeliinae	* Cathariostachys *
Bambuseae	PWB	Hickeliinae	* Decaryochloa *
Bambuseae	PWB	Hickeliinae	* Hickelia *
Bambuseae	PWB	Hickeliinae	* Hitchcockella *
Bambuseae	PWB	Hickeliinae	* Nastus *
Bambuseae	PWB	Hickeliinae	* Perrierbambus *
Bambuseae	PWB	Hickeliinae	* Sirochloa *
Bambuseae	PWB	Hickeliinae	* Sokinochloa *
Bambuseae	PWB	Hickeliinae	* Valiha *
Bambuseae	PWB	Holttumochloinae	* Holttumochloa *
Bambuseae	PWB	Holttumochloinae	* Kinabaluchloa *
Bambuseae	PWB	Holttumochloinae	* Nianhochloa *
Bambuseae	PWB	Melocanninae	* Annamocalamus *
Bambuseae	PWB	Melocanninae	* Cephalostachyum *
Bambuseae	PWB	Melocanninae	* Davidsea *
Bambuseae	PWB	Melocanninae	* Melocanna *
Bambuseae	PWB	Melocanninae	* Neohouzeaua *
Bambuseae	PWB	Melocanninae	* Ochlandra *
Bambuseae	PWB	Melocanninae	* Pseudostachyum *
Bambuseae	PWB	Melocanninae	* Schizostachyum *
Bambuseae	PWB	Melocanninae	* Stapletonia *
Bambuseae	PWB	Racemobambosinae	* Chloothamnus *
Bambuseae	PWB	Racemobambosinae	* Racemobambos *
Bambuseae	PWB	Racemobambosinae	* Widjajachloa *
Bambuseae	PWB	Temburongiinae	* Fimbribambusa *
Bambuseae	PWB	Temburongiinae	* Temburongia *
Arundinarieae	TWB	(incertae sedis)	* Khoonmengia *
Arundinarieae	TWB	Ampelocalaminae	* Ampelocalamus *
Arundinarieae	TWB	Ampelocalaminae	* Drepanostachyum *
Arundinarieae	TWB	Ampelocalaminae	* Himalayacalamus *
Arundinarieae	TWB	Arundinariinae	* Acidosasa *
Arundinarieae	TWB	Arundinariinae	* Arundinaria *
Arundinarieae	TWB	Arundinariinae	* Bashania *
Arundinarieae	TWB	Arundinariinae	* Brachystachyum *
Arundinarieae	TWB	Arundinariinae	* Chimonobambusa *
Arundinarieae	TWB	Arundinariinae	* Ferrocalamus *
Arundinarieae	TWB	Arundinariinae	* Gelidocalamus *
Arundinarieae	TWB	Arundinariinae	* Hibanobambusa *
Arundinarieae	TWB	Arundinariinae	* Indocalamus *
Arundinarieae	TWB	Arundinariinae	* Indosasa *
Arundinarieae	TWB	Arundinariinae	* Oligostachyum *
Arundinarieae	TWB	Arundinariinae	* Phyllostachys *
Arundinarieae	TWB	Arundinariinae	* Pleioblastus *
Arundinarieae	TWB	Arundinariinae	* Pseudosasa *
Arundinarieae	TWB	Arundinariinae	* Ravenochloa *
Arundinarieae	TWB	Arundinariinae	* Sasa *
Arundinarieae	TWB	Arundinariinae	* Sasaella *
Arundinarieae	TWB	Arundinariinae	* Sasamorpha *
Arundinarieae	TWB	Arundinariinae	* Semiarundinaria *
Arundinarieae	TWB	Arundinariinae	* Shibataea *
Arundinarieae	TWB	Arundinariinae	* Sinobambusa *
Arundinarieae	TWB	Arundinariinae	* Sinosasa *
Arundinarieae	TWB	Arundinariinae	* Vietnamocalamus *
Arundinarieae	TWB	Gaoligongshaniinae	* Gaoligongshania *
Arundinarieae	TWB	Hsuehochloinae	* Hsuehochloa *
Arundinarieae	TWB	Thamnocalaminae	* Bergbambos *
Arundinarieae	TWB	Thamnocalaminae	* Chimonocalamus *
Arundinarieae	TWB	Thamnocalaminae	* Fargesia *
Arundinarieae	TWB	Thamnocalaminae	* Kuruna *
Arundinarieae	TWB	Thamnocalaminae	* Oldeania *
Arundinarieae	TWB	Thamnocalaminae	* Sarocalamus *
Arundinarieae	TWB	Thamnocalaminae	* Thamnocalamus *
Arundinarieae	TWB	Thamnocalaminae	* Yushania *
