# Supplementary figures and images for: Global distribution models for the major bamboo (Poaceae, Bambusoideae) clades
Source: Biodivers Data J. 2025 Aug 26;13:e153436. doi: 10.3897/BDJ.13.e153436 (PMC12405939; doi:10.3897/BDJ.13.e153436)

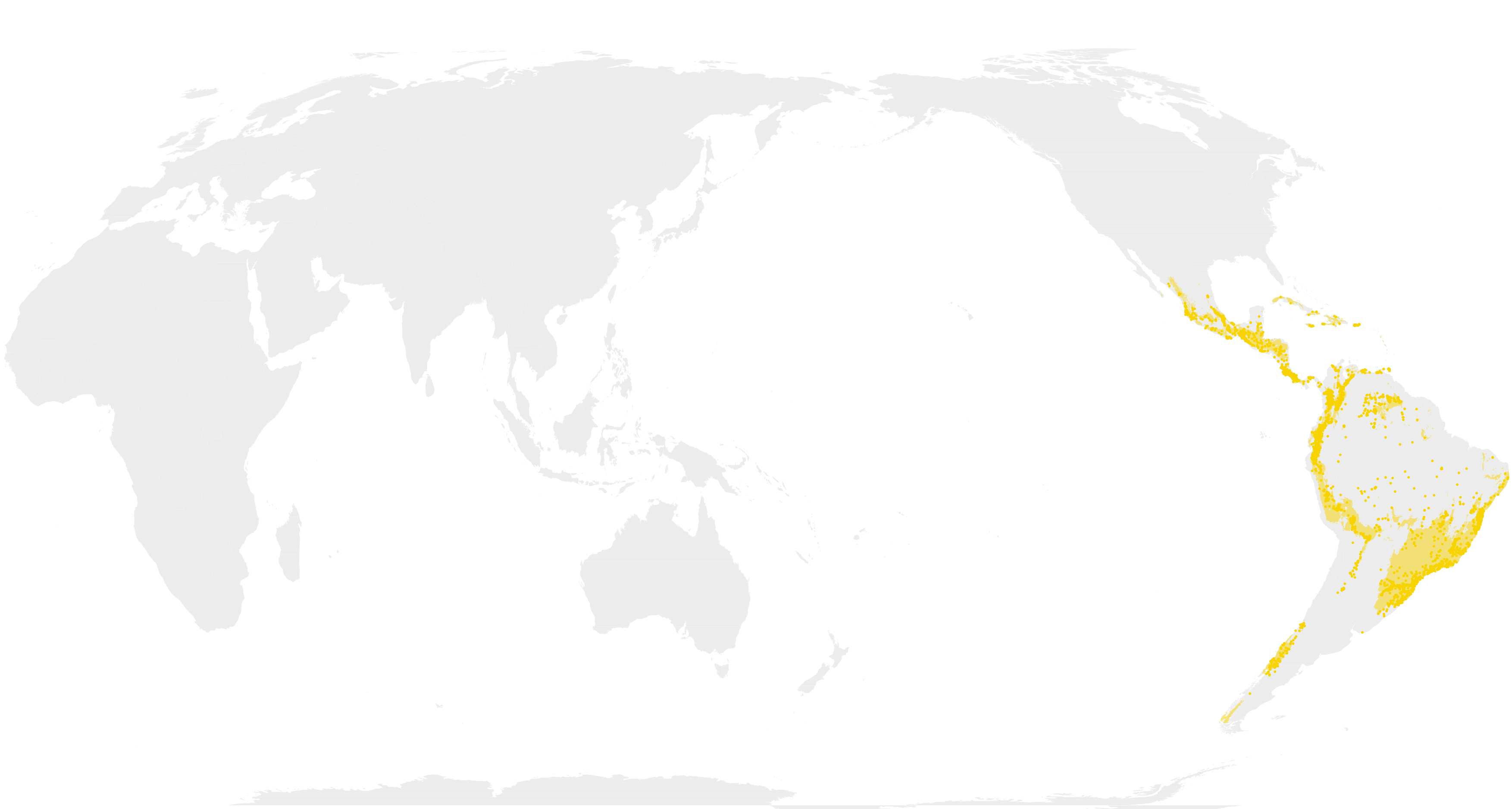

Supplement: Supplementary material 5 — Map images (png) of estimated Bambusoideae clade distributions [file bdj-13-e153436-s005.zip › Suppl. 4 - GIS Output Images/pacific centered/points+areas/pacific-nwb-points+areas.png]

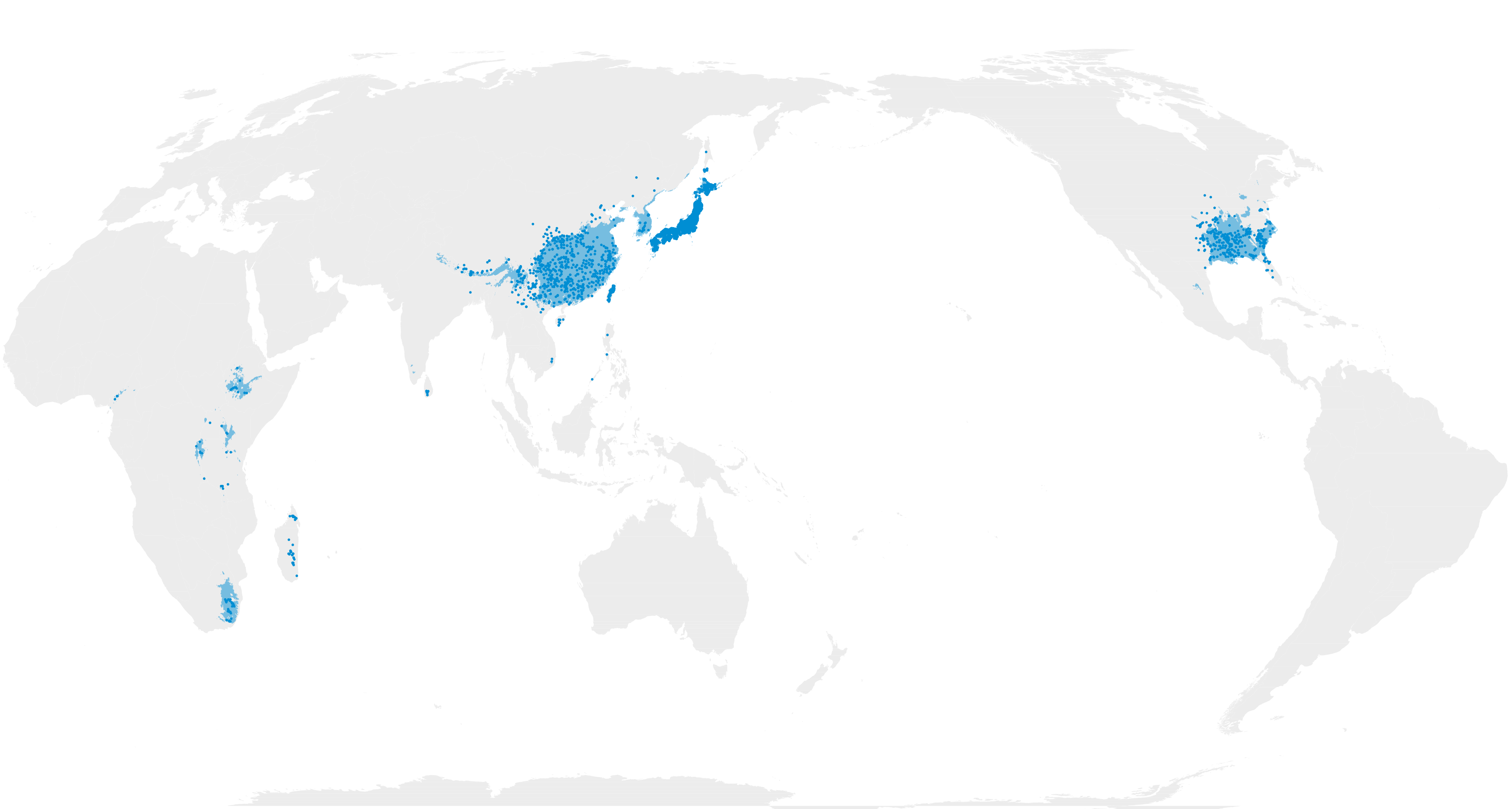

Supplement: Supplementary material 5 — Map images (png) of estimated Bambusoideae clade distributions [file bdj-13-e153436-s005.zip › Suppl. 4 - GIS Output Images/pacific centered/points+areas/pacific-twb-points+areas.png]

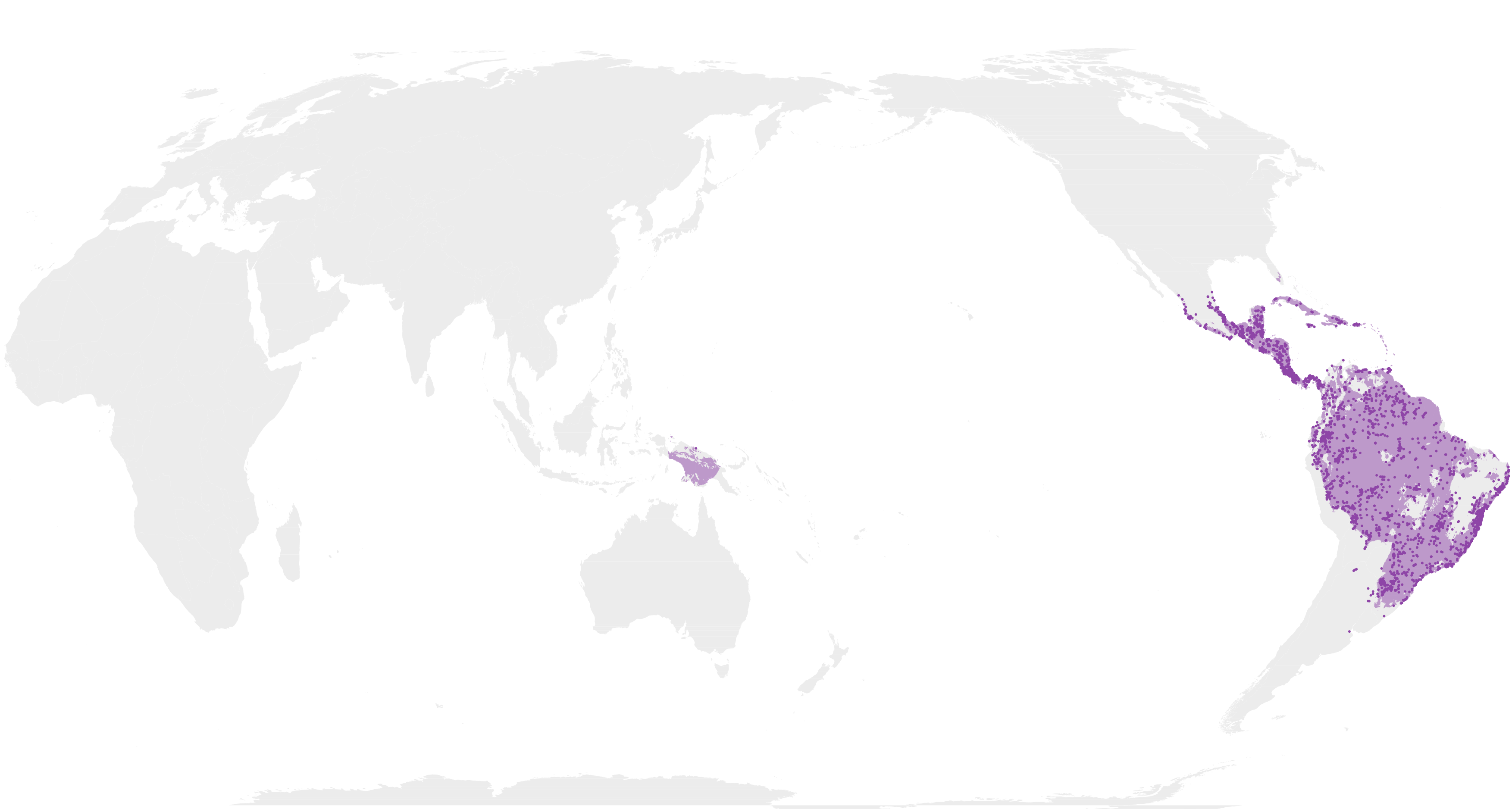

Supplement: Supplementary material 5 — Map images (png) of estimated Bambusoideae clade distributions [file bdj-13-e153436-s005.zip › Suppl. 4 - GIS Output Images/pacific centered/points+areas/pacific-herbaceous-points+areas.png]

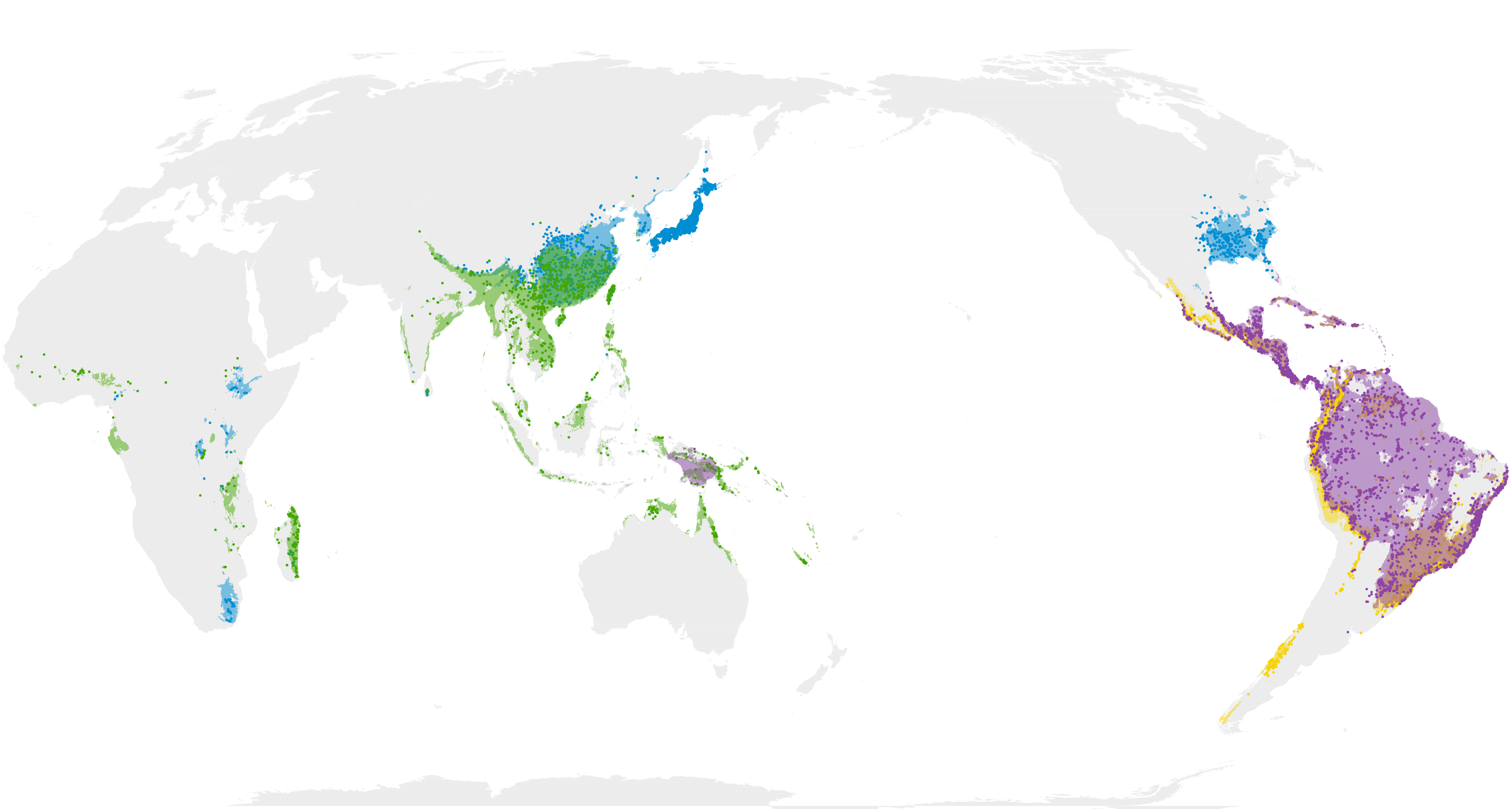

Supplement: Supplementary material 5 — Map images (png) of estimated Bambusoideae clade distributions [file bdj-13-e153436-s005.zip › Suppl. 4 - GIS Output Images/pacific centered/points+areas/pacific-all-points+areas.png]

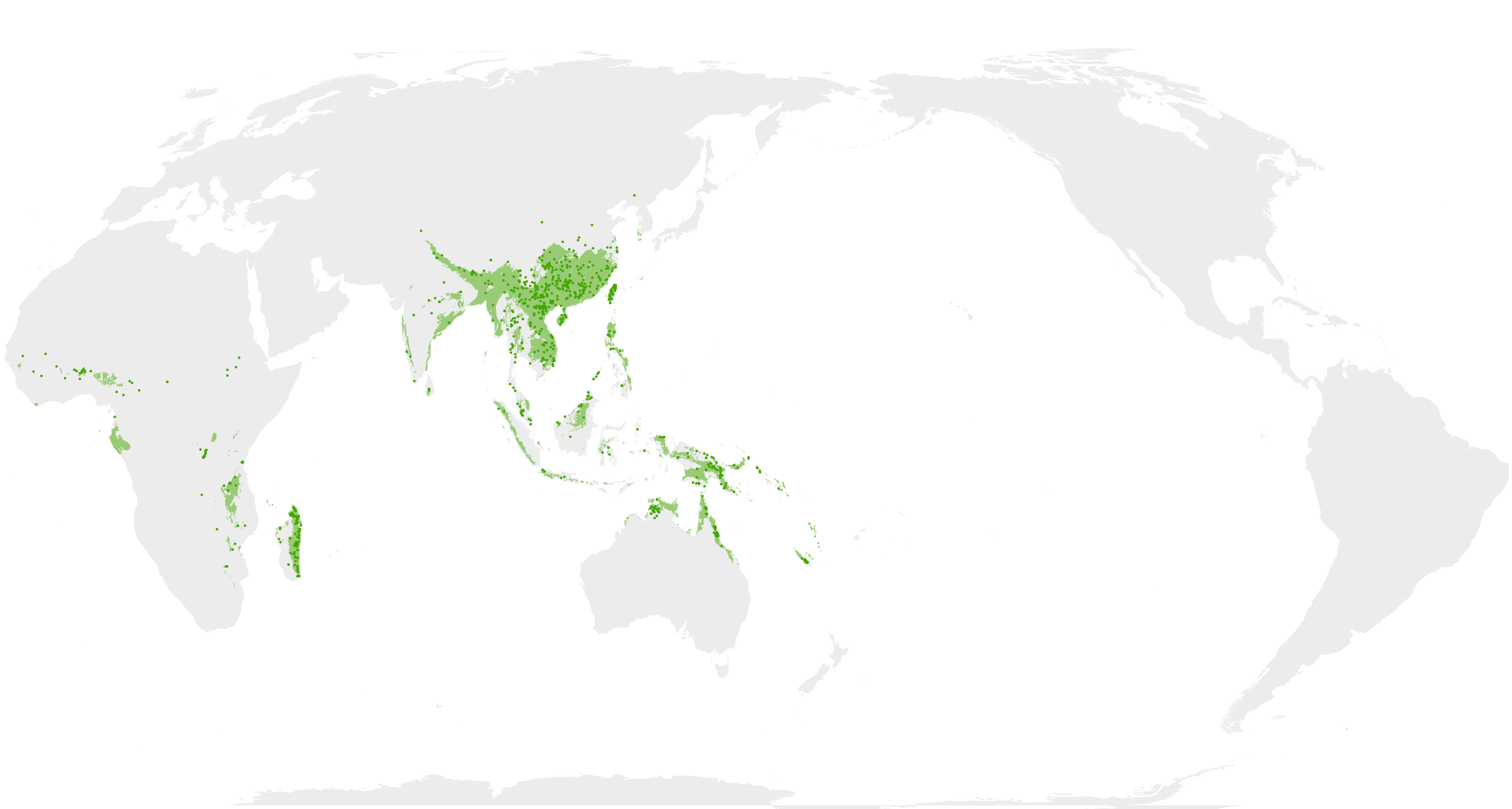

Supplement: Supplementary material 5 — Map images (png) of estimated Bambusoideae clade distributions [file bdj-13-e153436-s005.zip › Suppl. 4 - GIS Output Images/pacific centered/points+areas/pacific-pwb-points+areas.png]

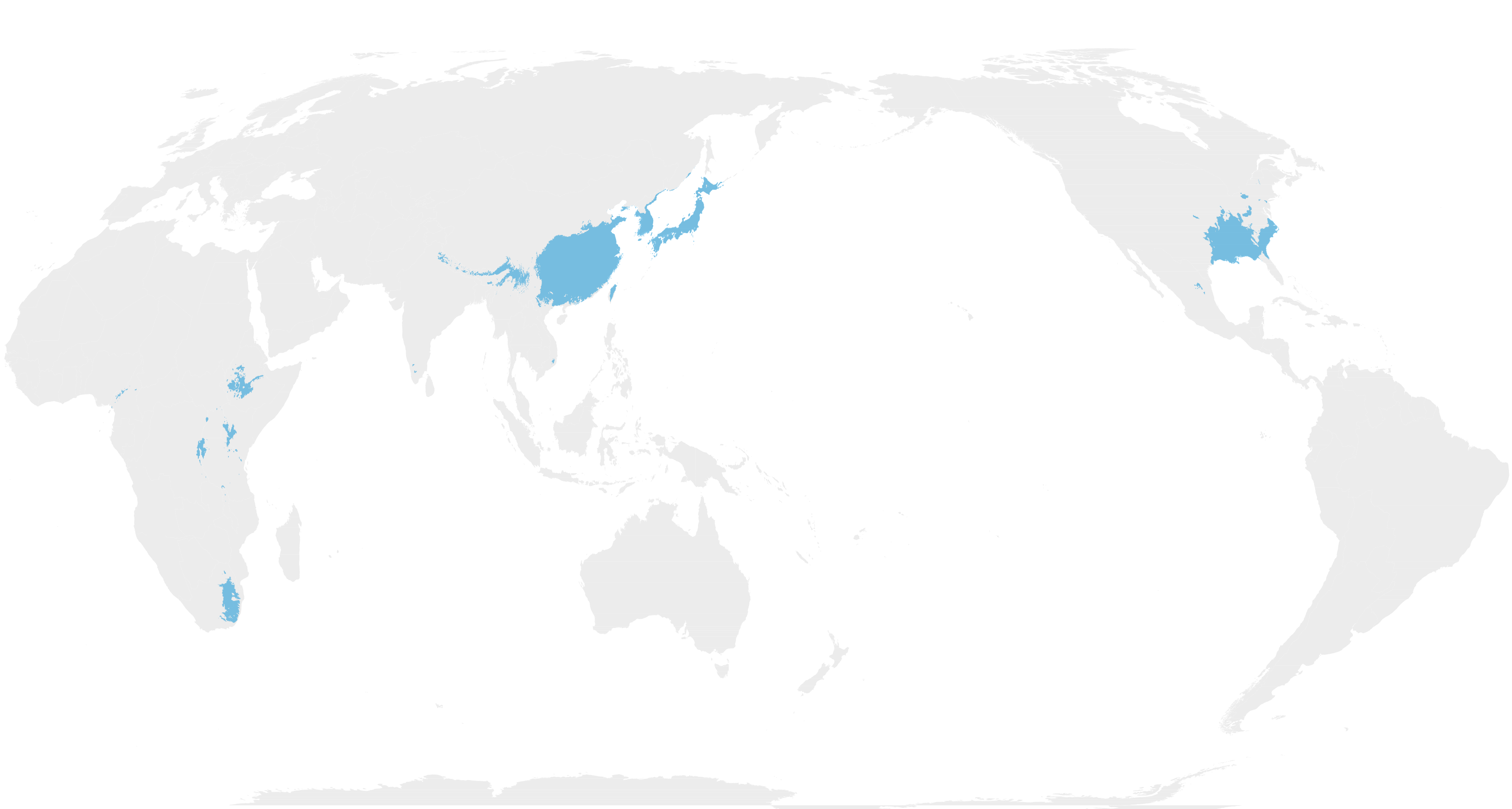

Supplement: Supplementary material 5 — Map images (png) of estimated Bambusoideae clade distributions [file bdj-13-e153436-s005.zip › Suppl. 4 - GIS Output Images/pacific centered/areas/pacific-twb-areas.png]

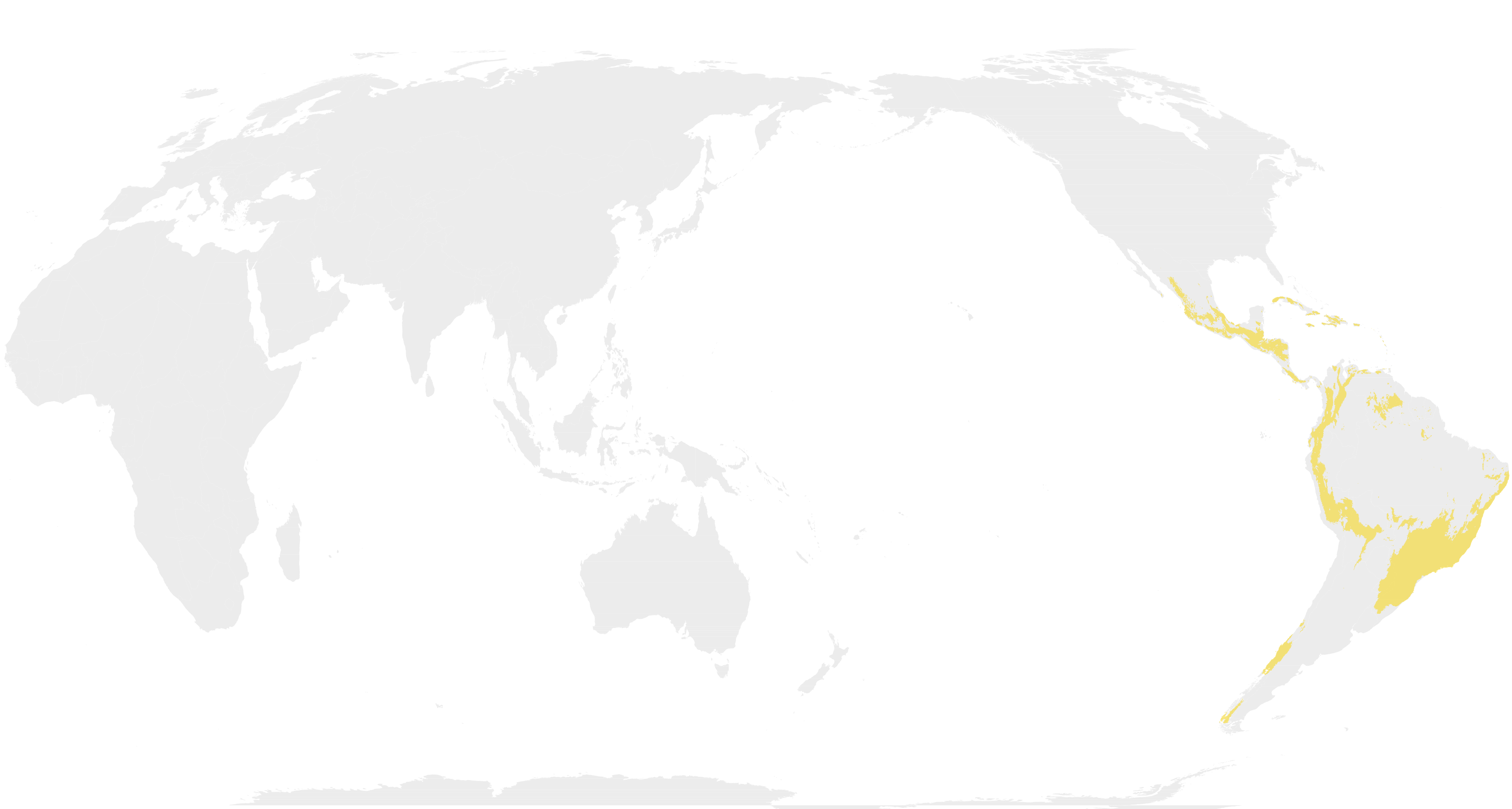

Supplement: Supplementary material 5 — Map images (png) of estimated Bambusoideae clade distributions [file bdj-13-e153436-s005.zip › Suppl. 4 - GIS Output Images/pacific centered/areas/pacific-nwb-areas.png]

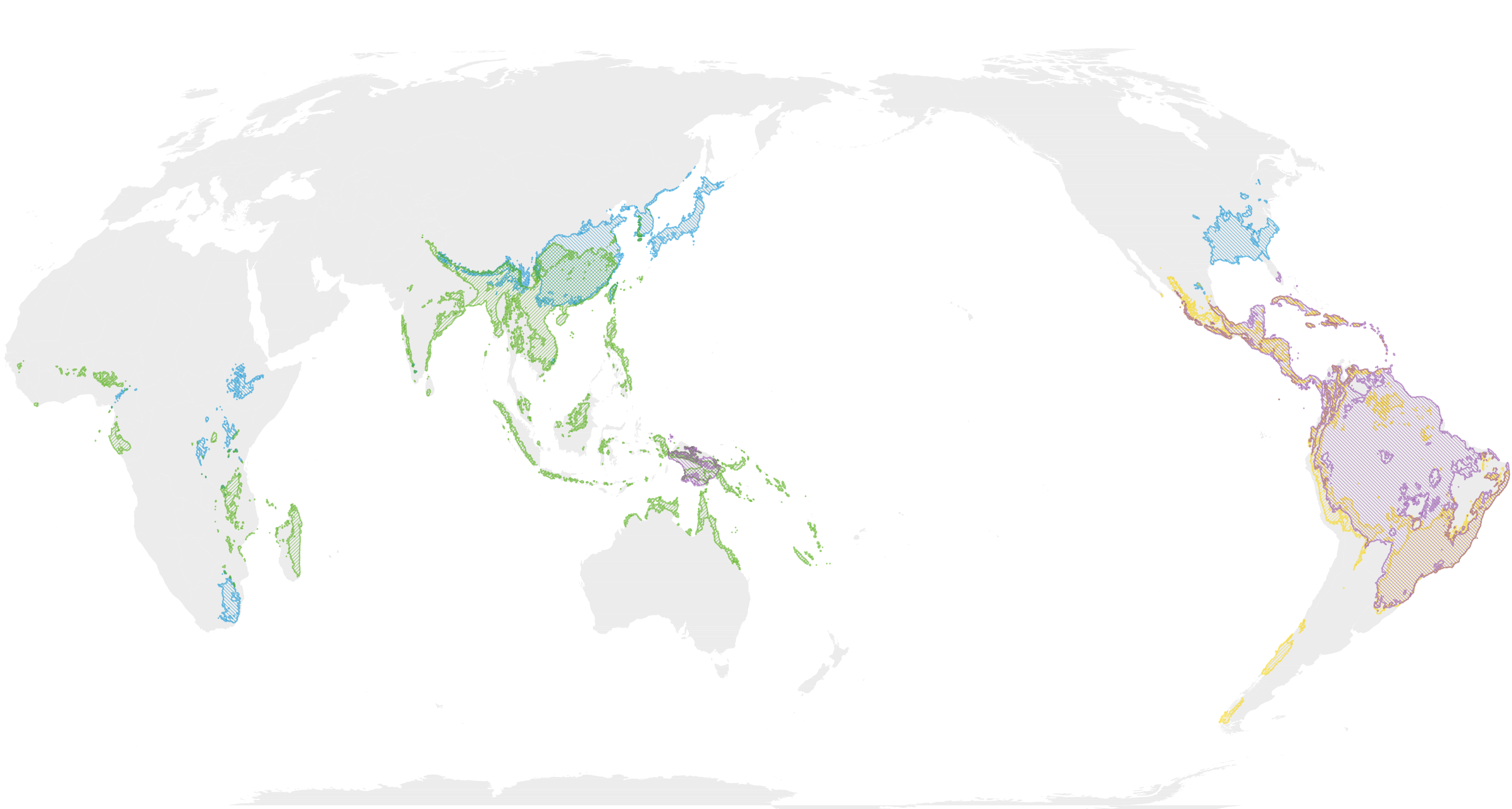

Supplement: Supplementary material 5 — Map images (png) of estimated Bambusoideae clade distributions [file bdj-13-e153436-s005.zip › Suppl. 4 - GIS Output Images/pacific centered/areas/pacific-all-crosshatched.png]

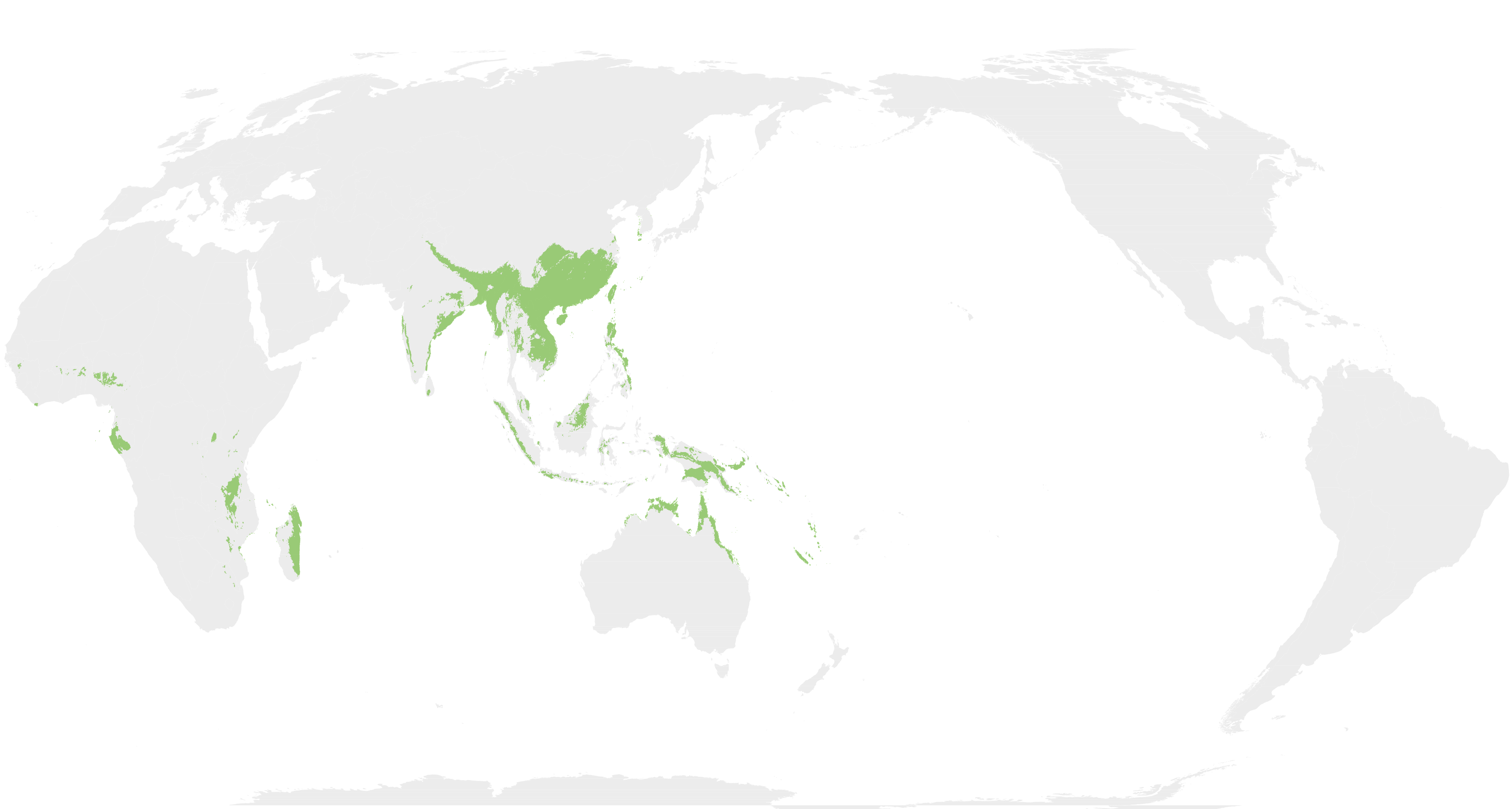

Supplement: Supplementary material 5 — Map images (png) of estimated Bambusoideae clade distributions [file bdj-13-e153436-s005.zip › Suppl. 4 - GIS Output Images/pacific centered/areas/pacific-pwb-areas.png]

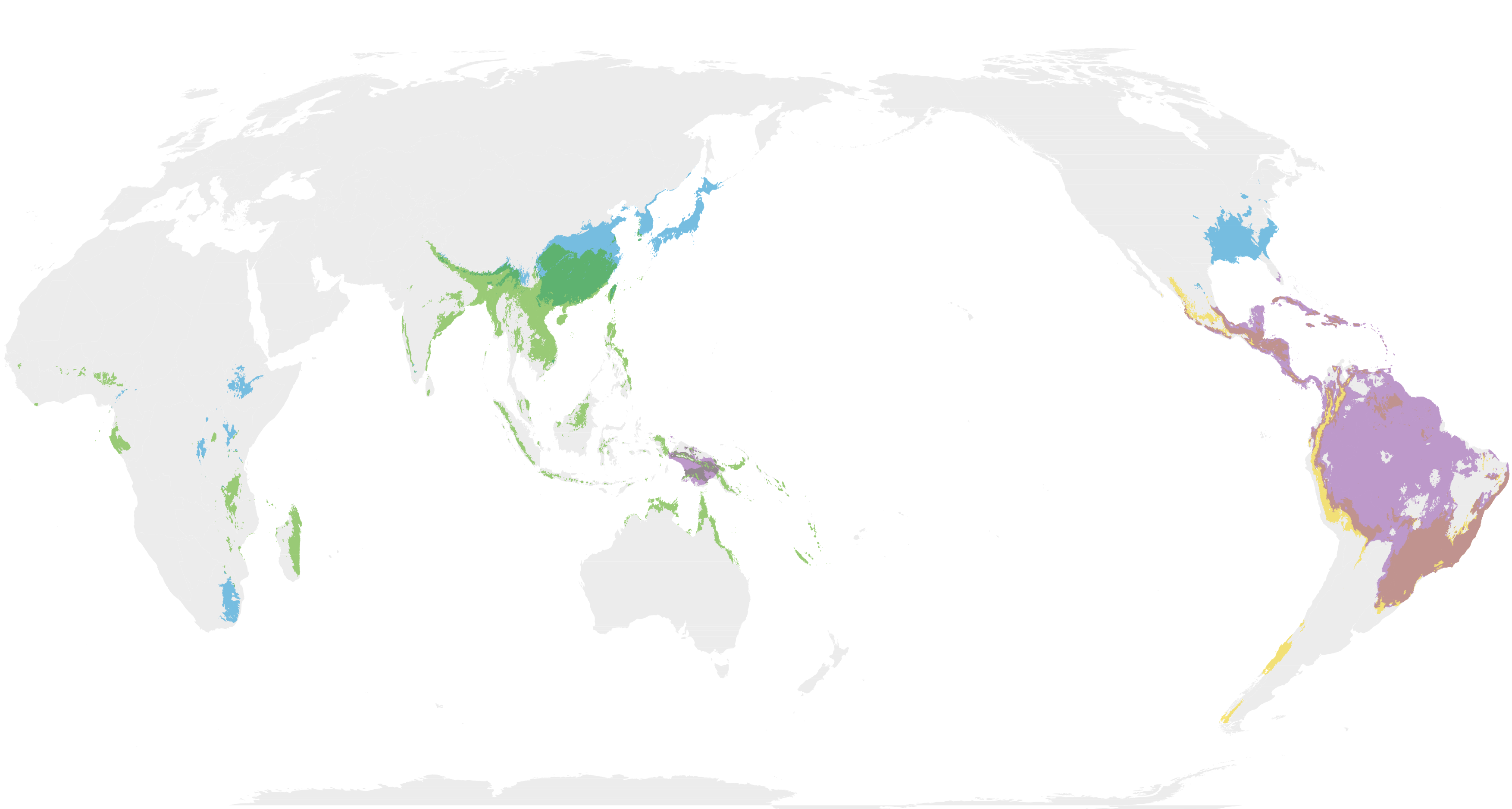

Supplement: Supplementary material 5 — Map images (png) of estimated Bambusoideae clade distributions [file bdj-13-e153436-s005.zip › Suppl. 4 - GIS Output Images/pacific centered/areas/pacific-all-areas.png]

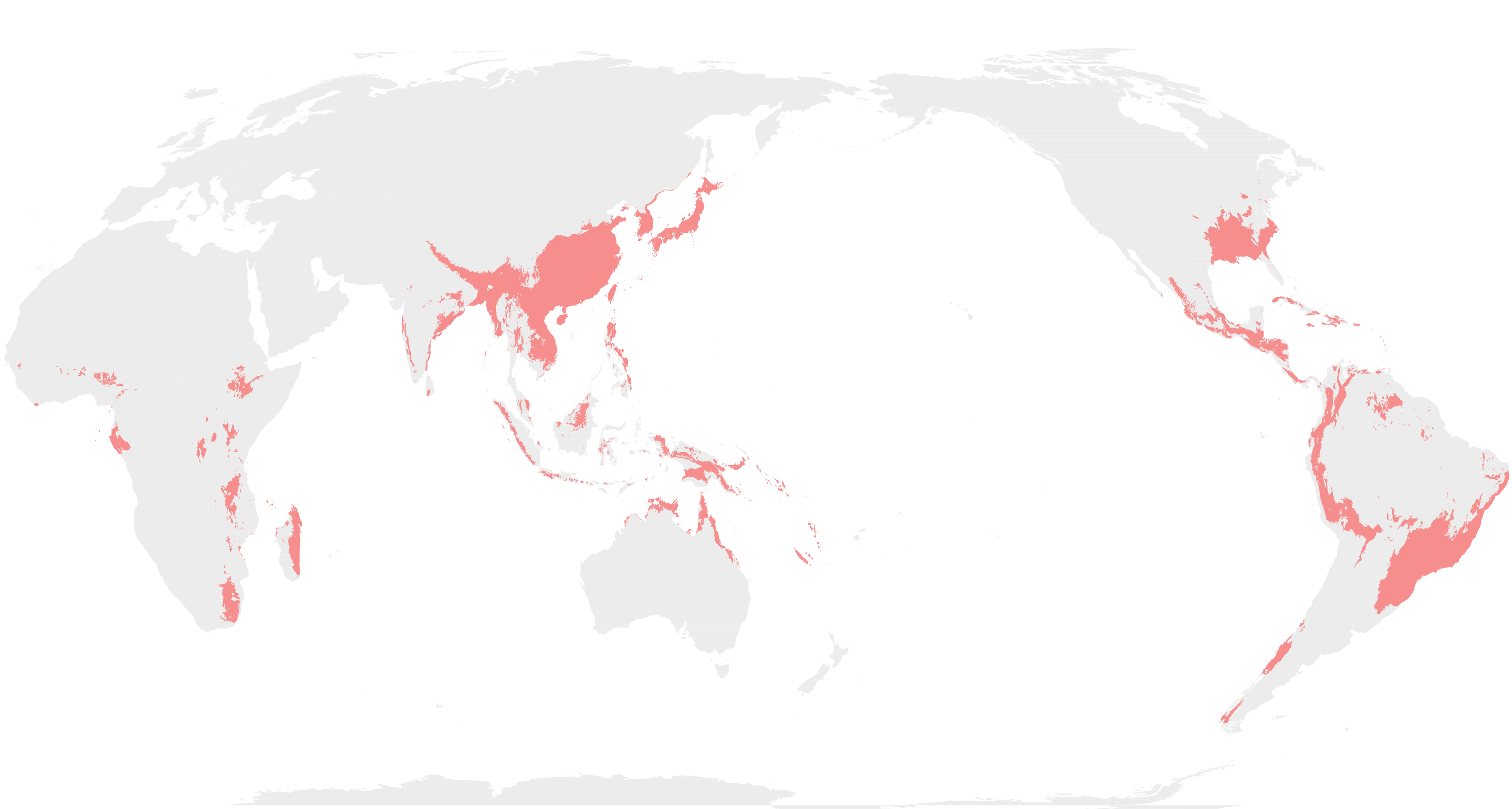

Supplement: Supplementary material 5 — Map images (png) of estimated Bambusoideae clade distributions [file bdj-13-e153436-s005.zip › Suppl. 4 - GIS Output Images/pacific centered/areas/pacific-woody-areas.png]

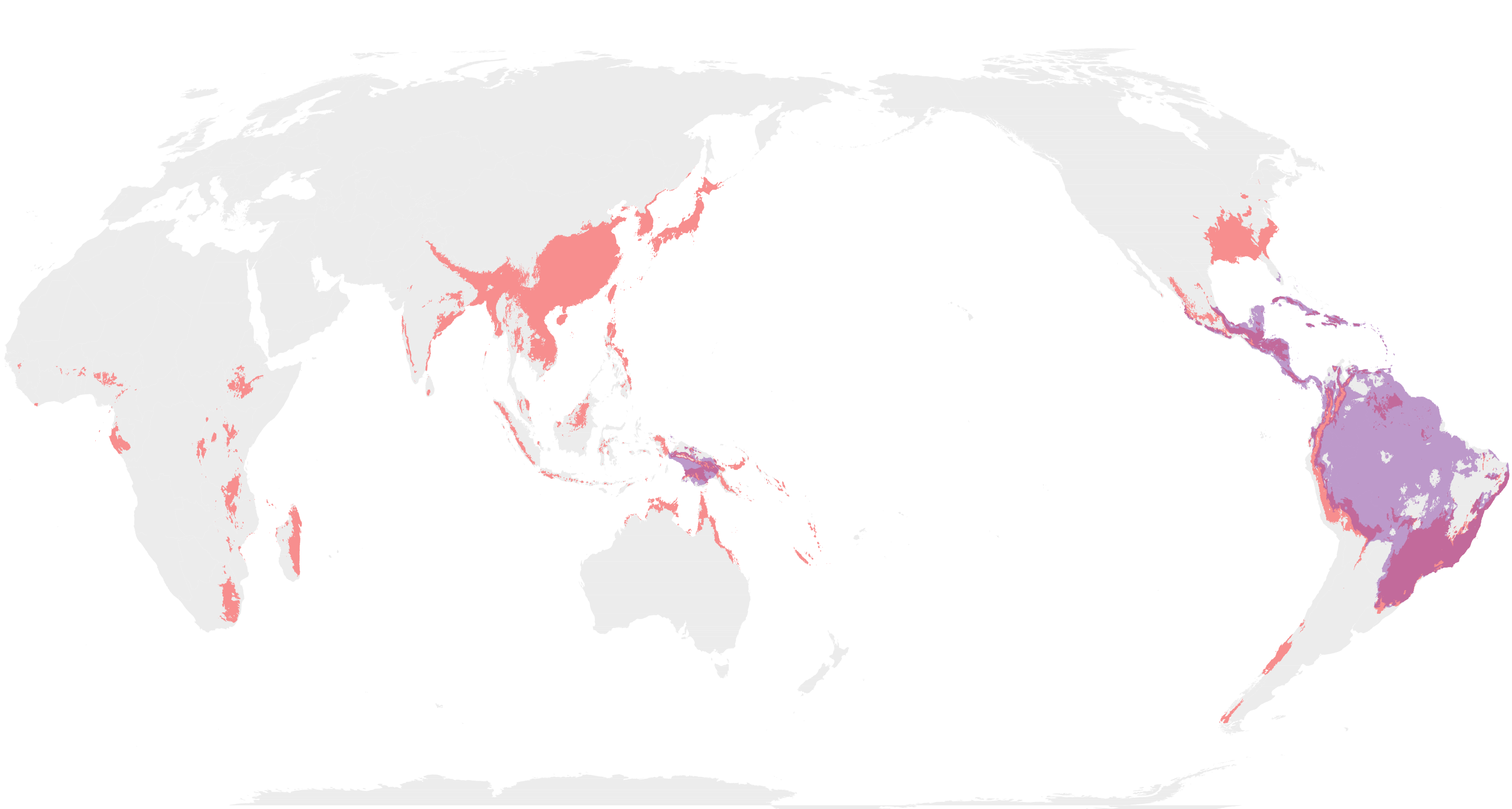

Supplement: Supplementary material 5 — Map images (png) of estimated Bambusoideae clade distributions [file bdj-13-e153436-s005.zip › Suppl. 4 - GIS Output Images/pacific centered/areas/pacific-woody_vs_herbaceous-areas.png]

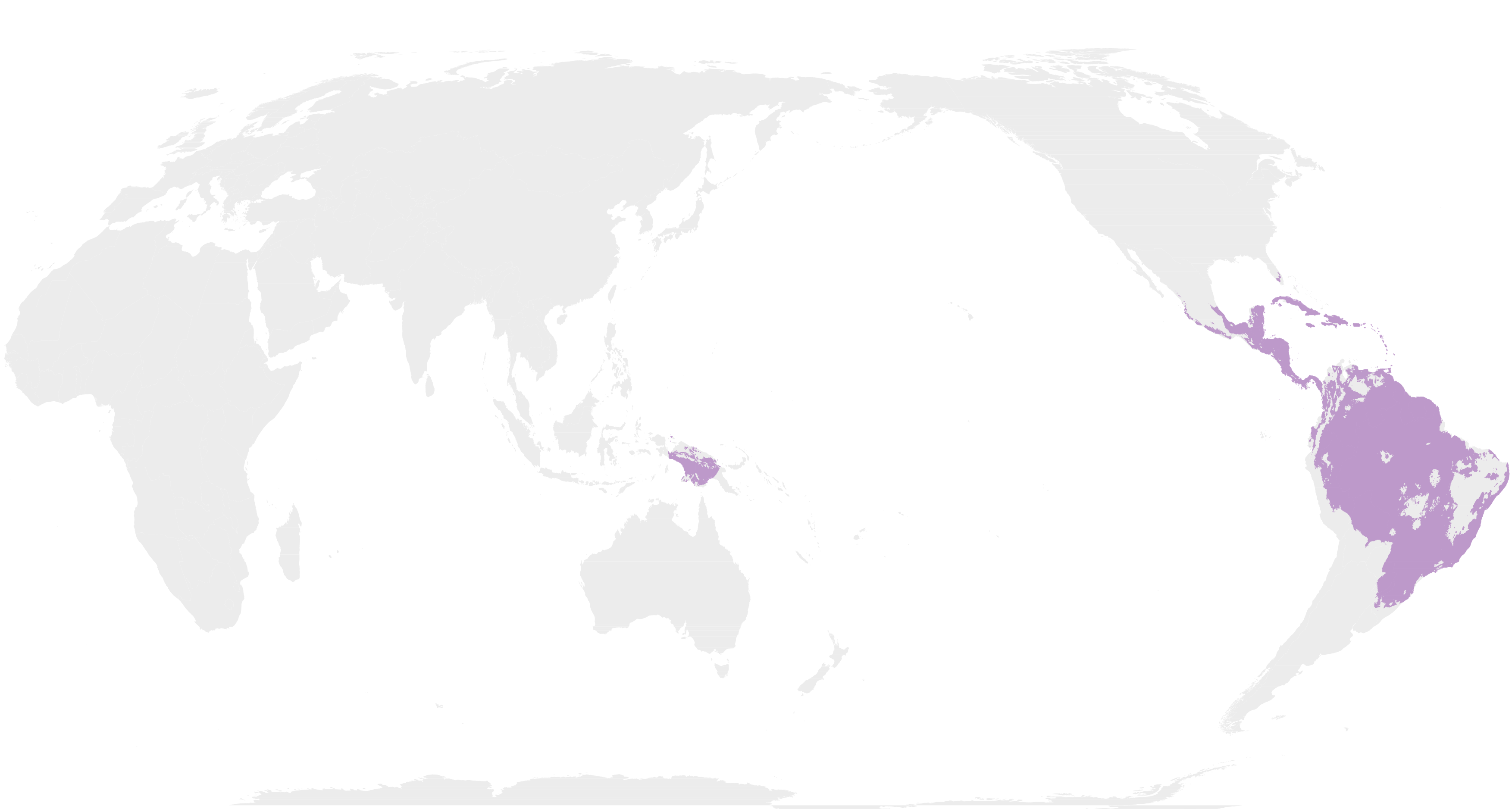

Supplement: Supplementary material 5 — Map images (png) of estimated Bambusoideae clade distributions [file bdj-13-e153436-s005.zip › Suppl. 4 - GIS Output Images/pacific centered/areas/pacific-herbaceous-areas.png]

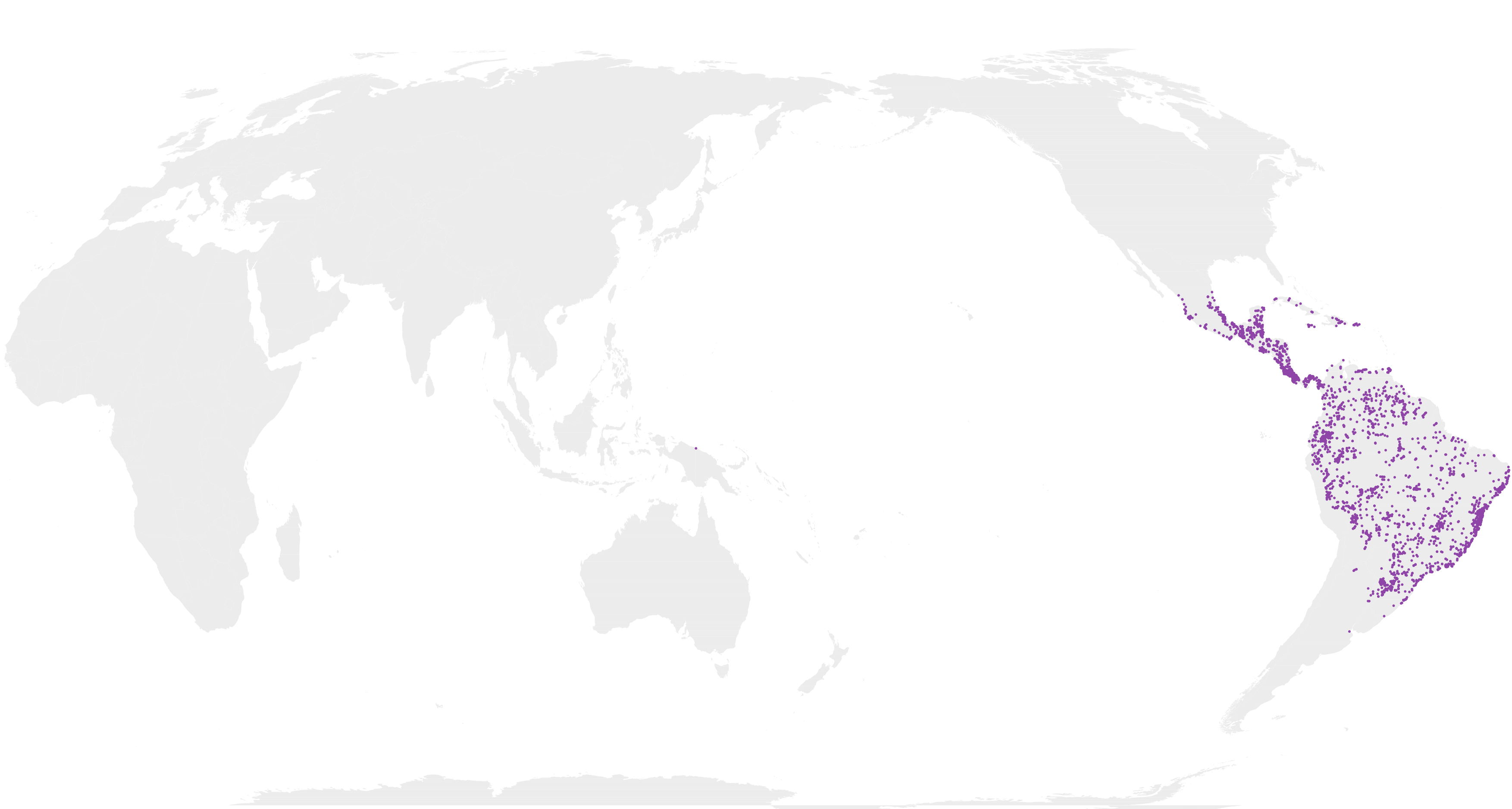

Supplement: Supplementary material 5 — Map images (png) of estimated Bambusoideae clade distributions [file bdj-13-e153436-s005.zip › Suppl. 4 - GIS Output Images/pacific centered/points/pacific-herbaceous-points.png]

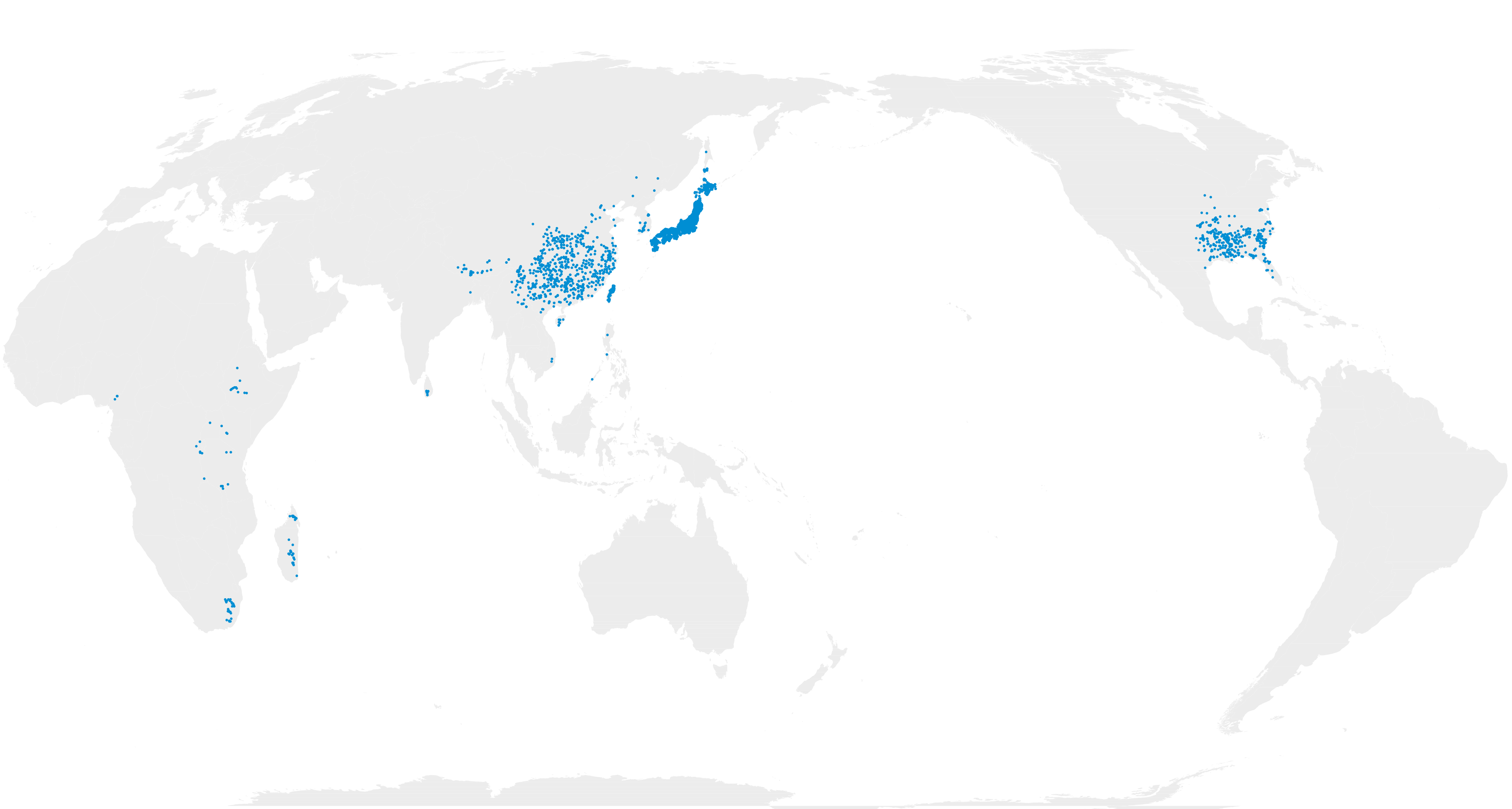

Supplement: Supplementary material 5 — Map images (png) of estimated Bambusoideae clade distributions [file bdj-13-e153436-s005.zip › Suppl. 4 - GIS Output Images/pacific centered/points/pacific-twb-points.png]

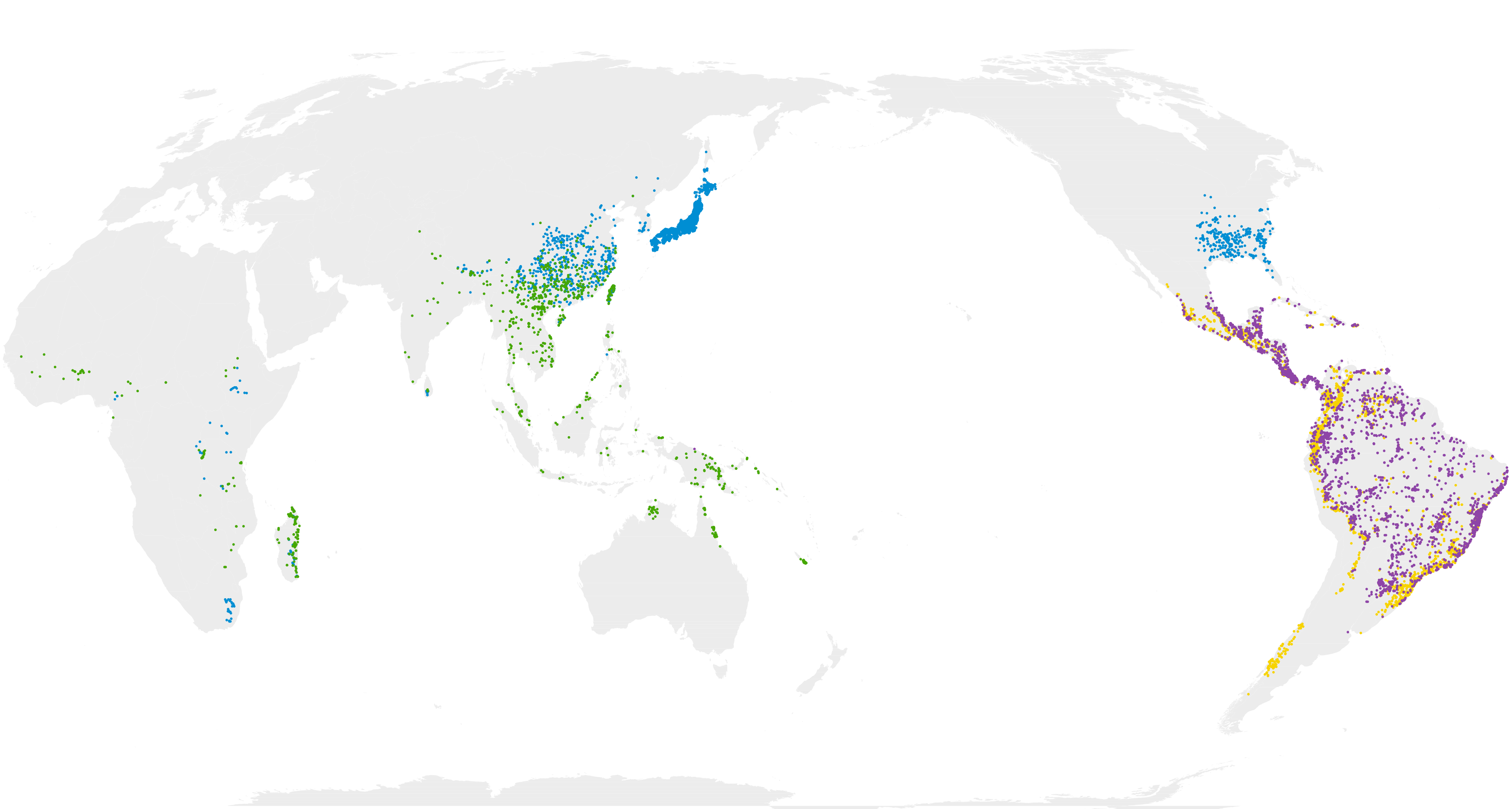

Supplement: Supplementary material 5 — Map images (png) of estimated Bambusoideae clade distributions [file bdj-13-e153436-s005.zip › Suppl. 4 - GIS Output Images/pacific centered/points/pacific-all-points.png]

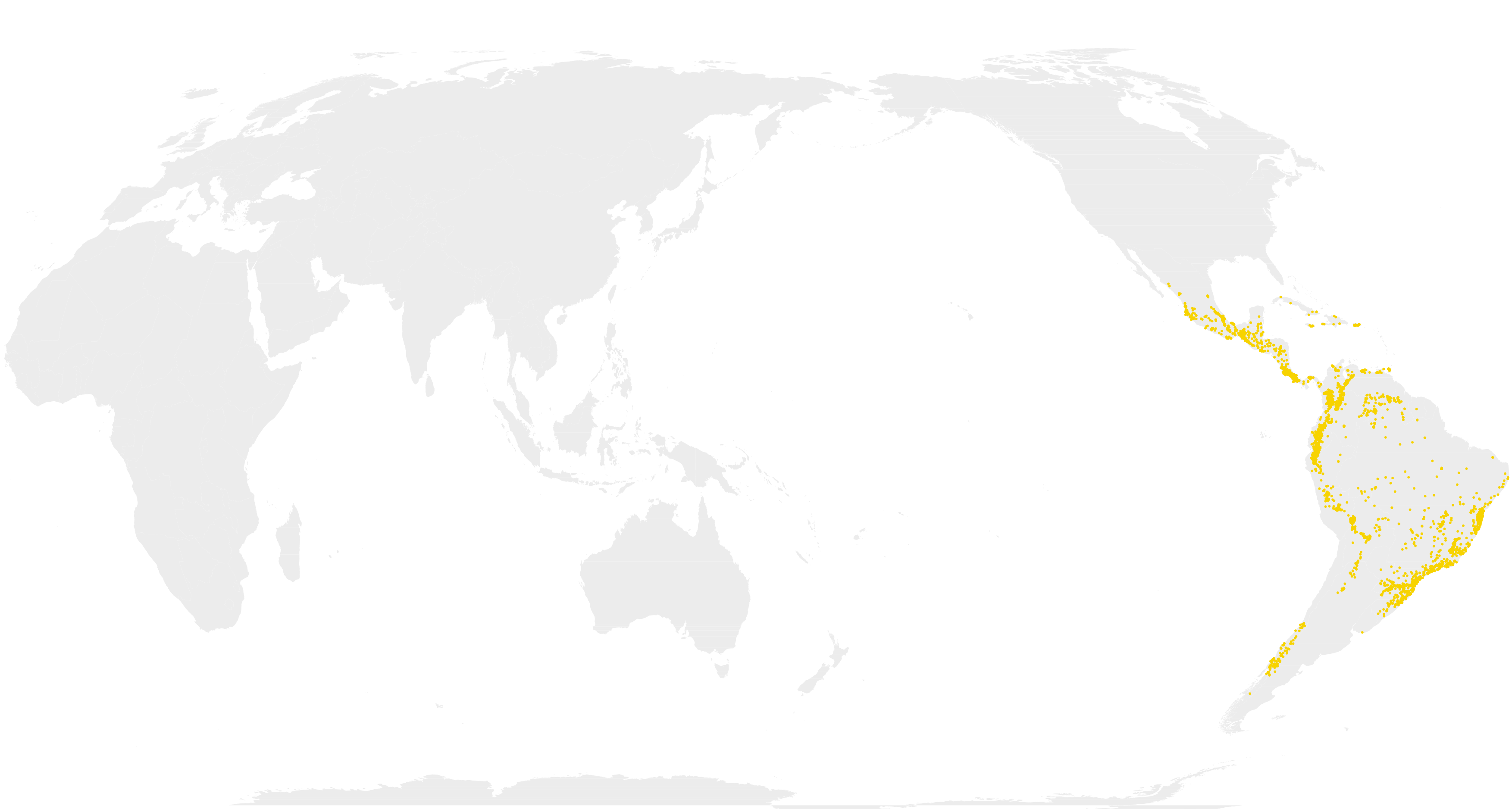

Supplement: Supplementary material 5 — Map images (png) of estimated Bambusoideae clade distributions [file bdj-13-e153436-s005.zip › Suppl. 4 - GIS Output Images/pacific centered/points/pacific-nwb-points.png]

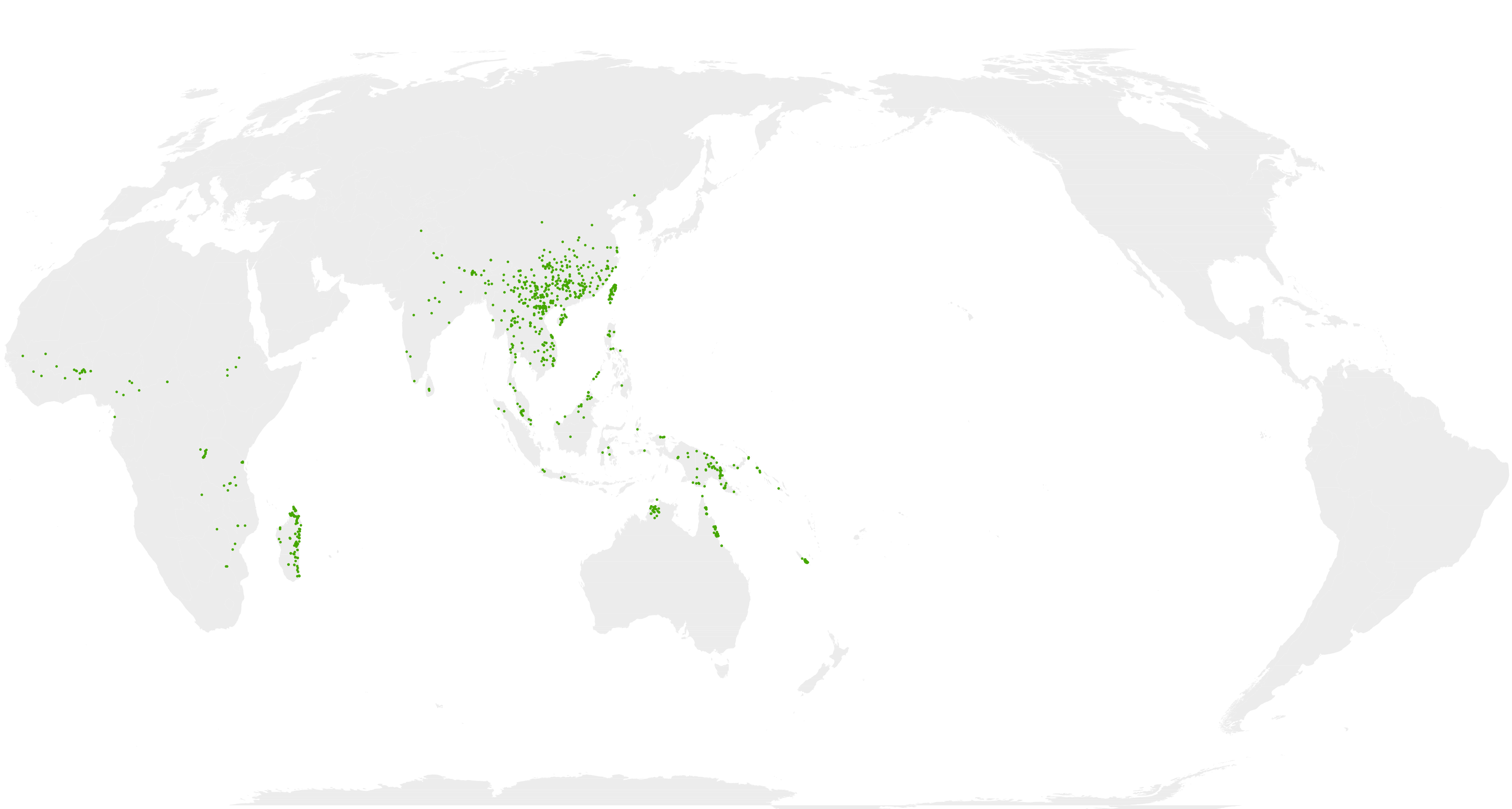

Supplement: Supplementary material 5 — Map images (png) of estimated Bambusoideae clade distributions [file bdj-13-e153436-s005.zip › Suppl. 4 - GIS Output Images/pacific centered/points/pacific-pwb-points.png]

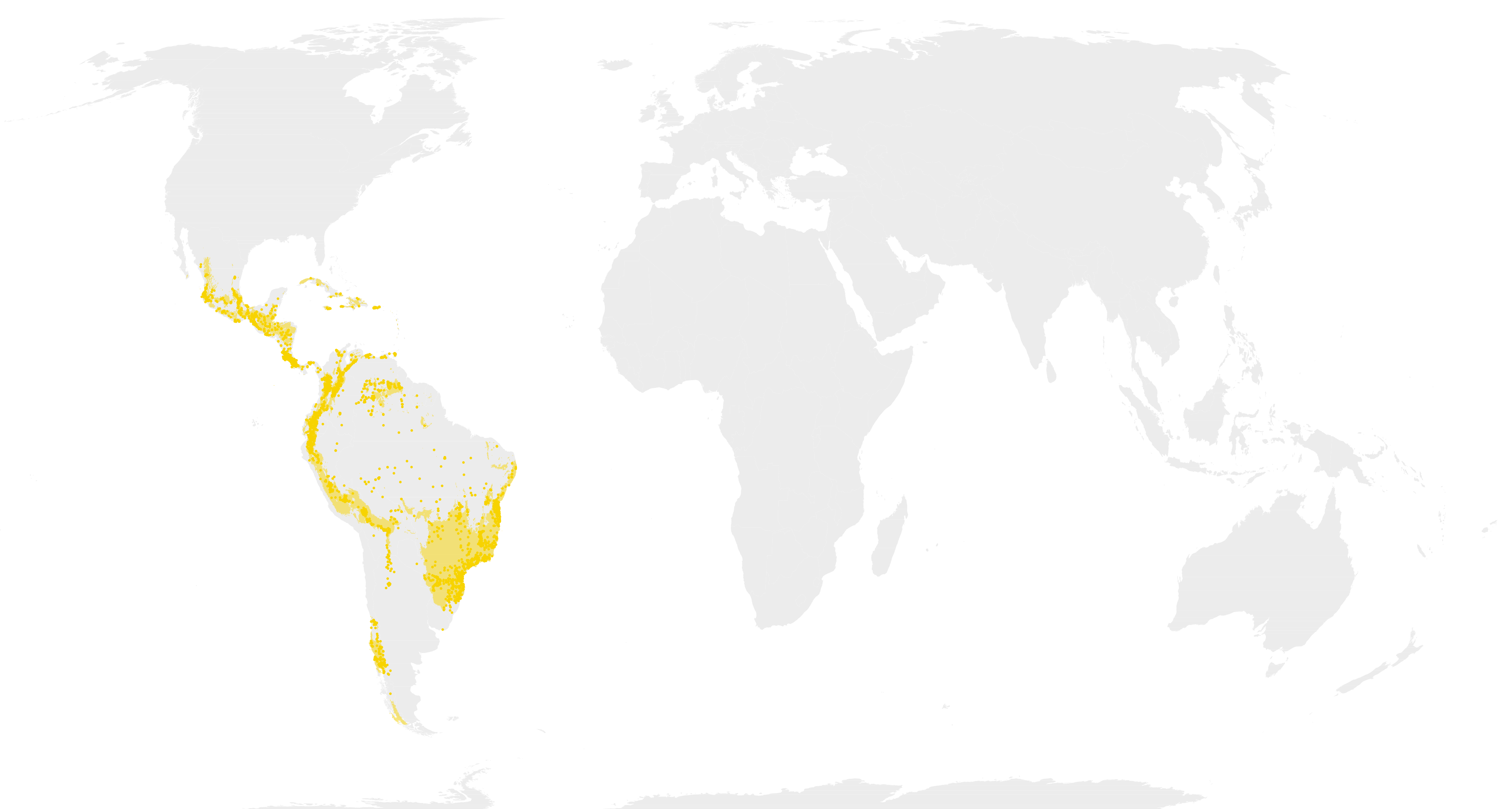

Supplement: Supplementary material 5 — Map images (png) of estimated Bambusoideae clade distributions [file bdj-13-e153436-s005.zip › Suppl. 4 - GIS Output Images/atlantic centered/points+areas/atlantic-nwb-points+areas.png]

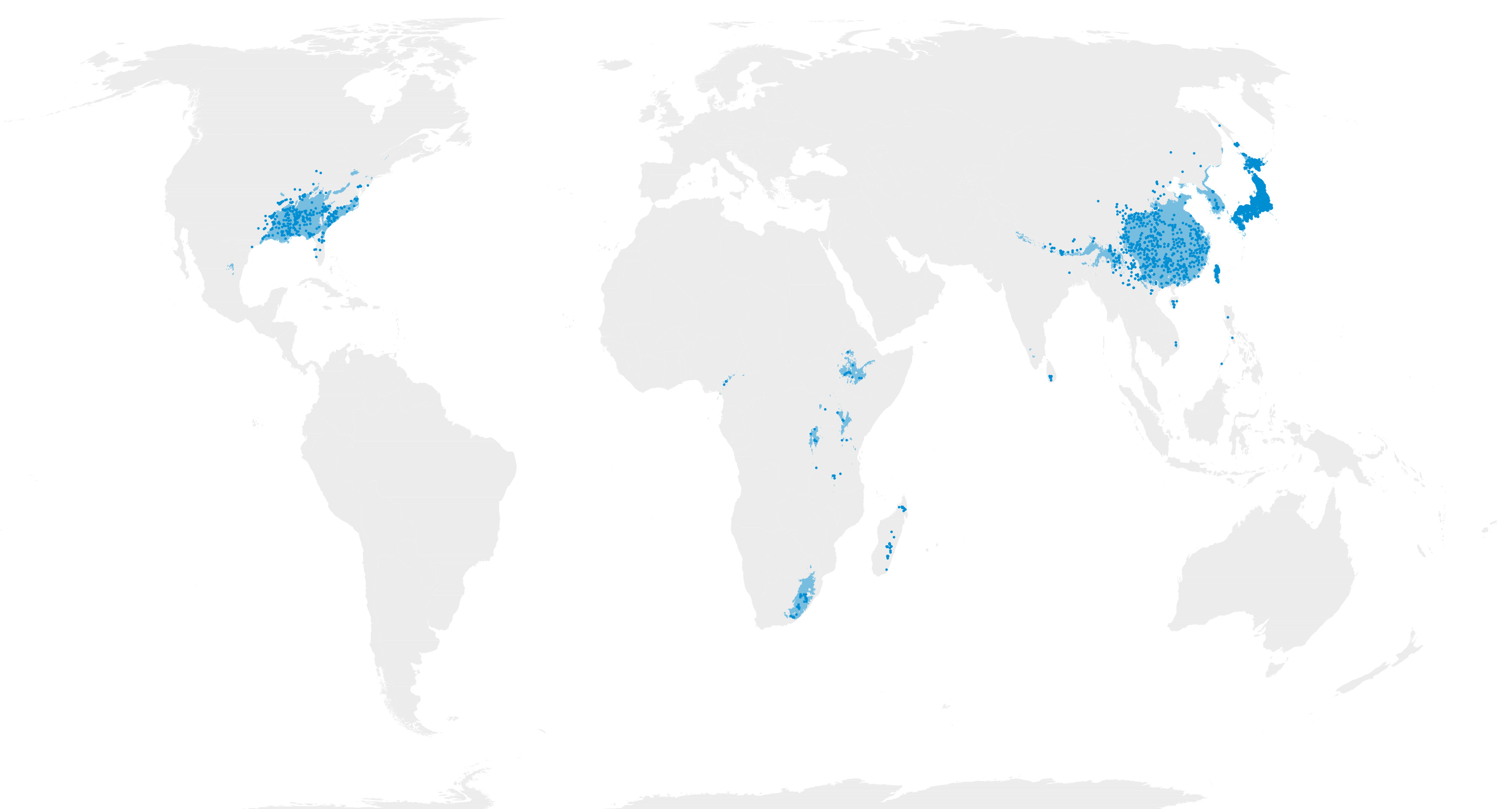

Supplement: Supplementary material 5 — Map images (png) of estimated Bambusoideae clade distributions [file bdj-13-e153436-s005.zip › Suppl. 4 - GIS Output Images/atlantic centered/points+areas/atlantic-twb-points+areas.png]

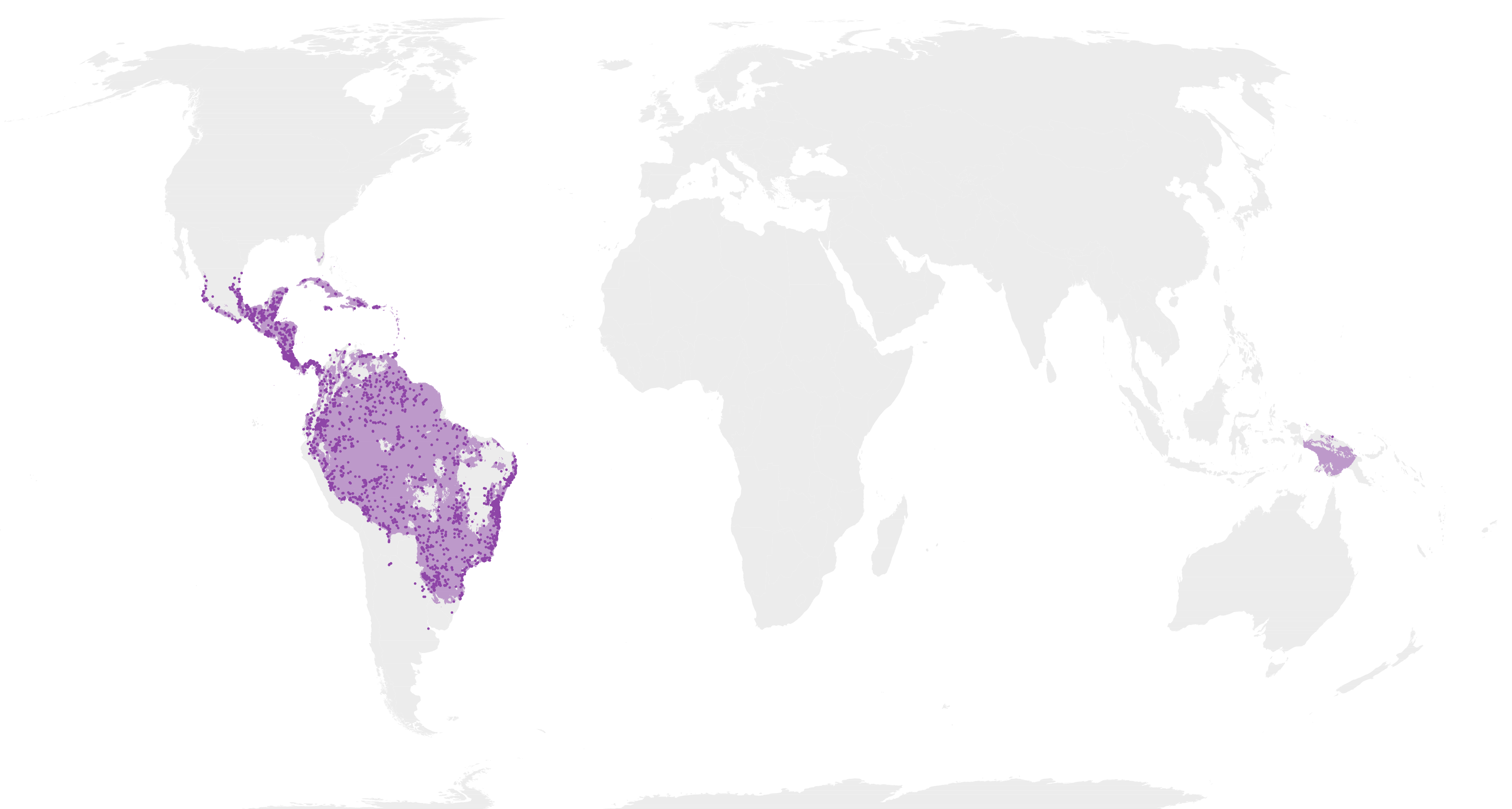

Supplement: Supplementary material 5 — Map images (png) of estimated Bambusoideae clade distributions [file bdj-13-e153436-s005.zip › Suppl. 4 - GIS Output Images/atlantic centered/points+areas/atlantic-herbaceous-points+areas.png]

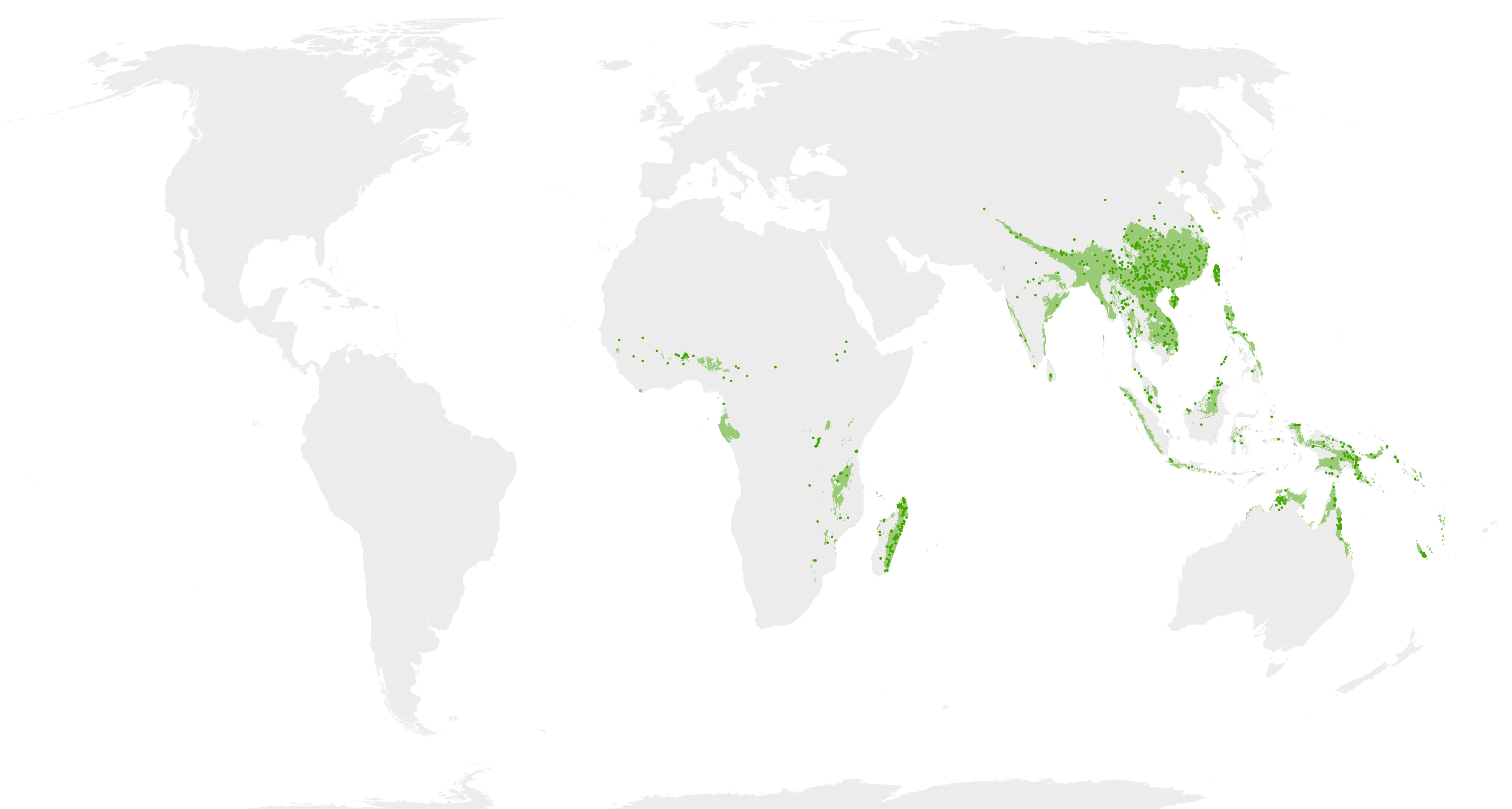

Supplement: Supplementary material 5 — Map images (png) of estimated Bambusoideae clade distributions [file bdj-13-e153436-s005.zip › Suppl. 4 - GIS Output Images/atlantic centered/points+areas/atlantic-pwb-points+areas.png]

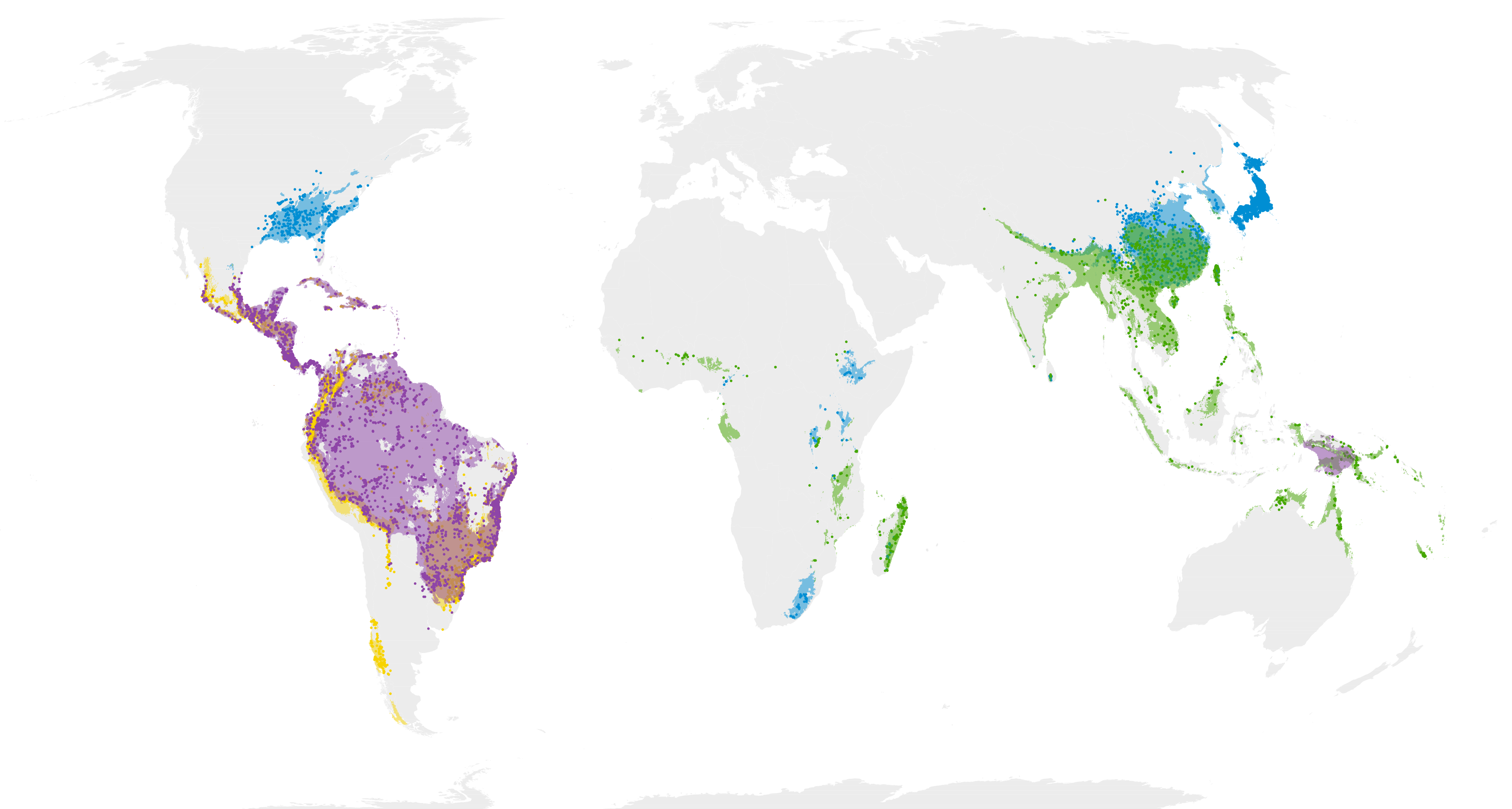

Supplement: Supplementary material 5 — Map images (png) of estimated Bambusoideae clade distributions [file bdj-13-e153436-s005.zip › Suppl. 4 - GIS Output Images/atlantic centered/points+areas/atlantic-all-points+areas.png]

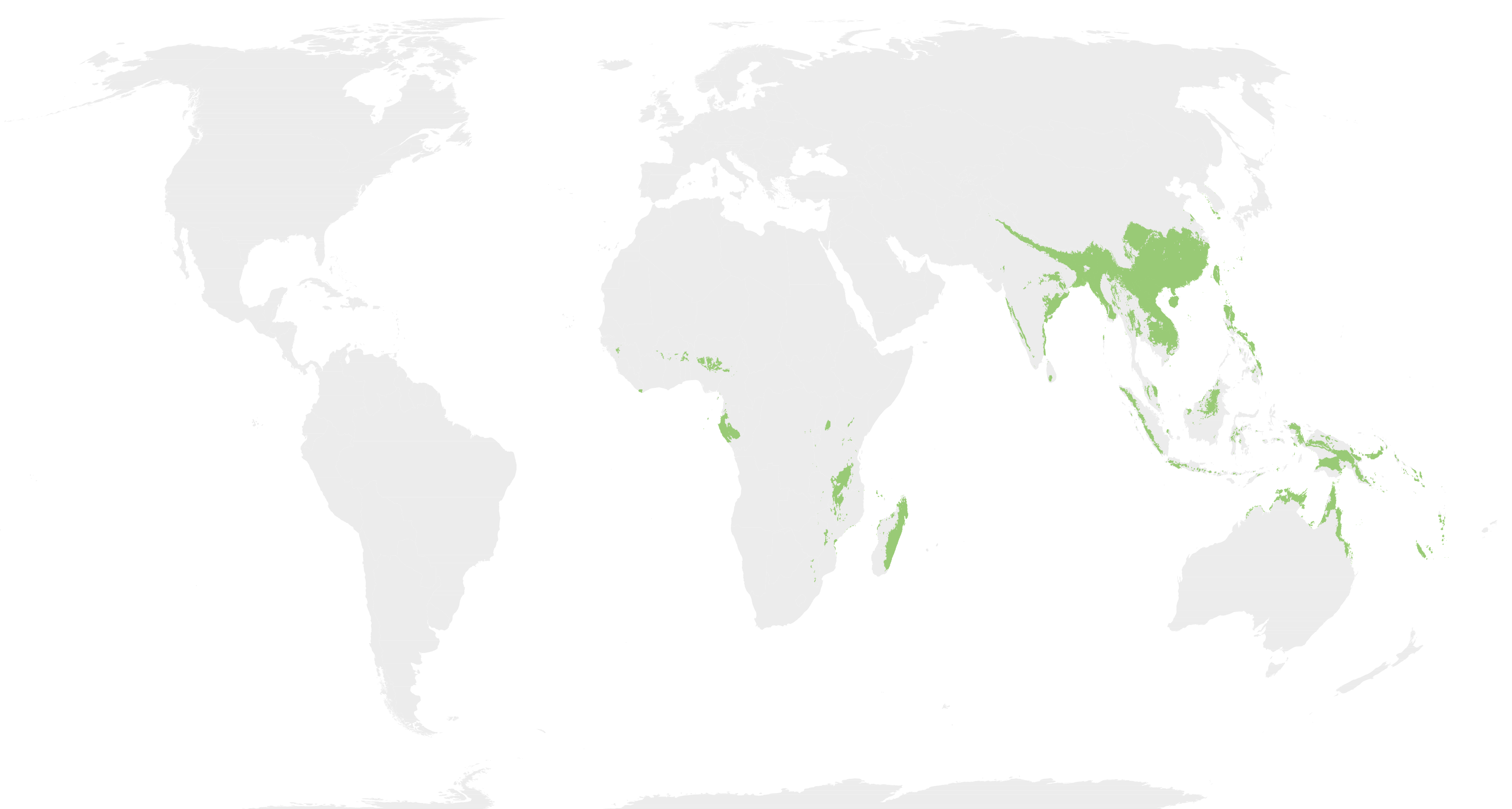

Supplement: Supplementary material 5 — Map images (png) of estimated Bambusoideae clade distributions [file bdj-13-e153436-s005.zip › Suppl. 4 - GIS Output Images/atlantic centered/areas/atlantic-pwb-areas.png]

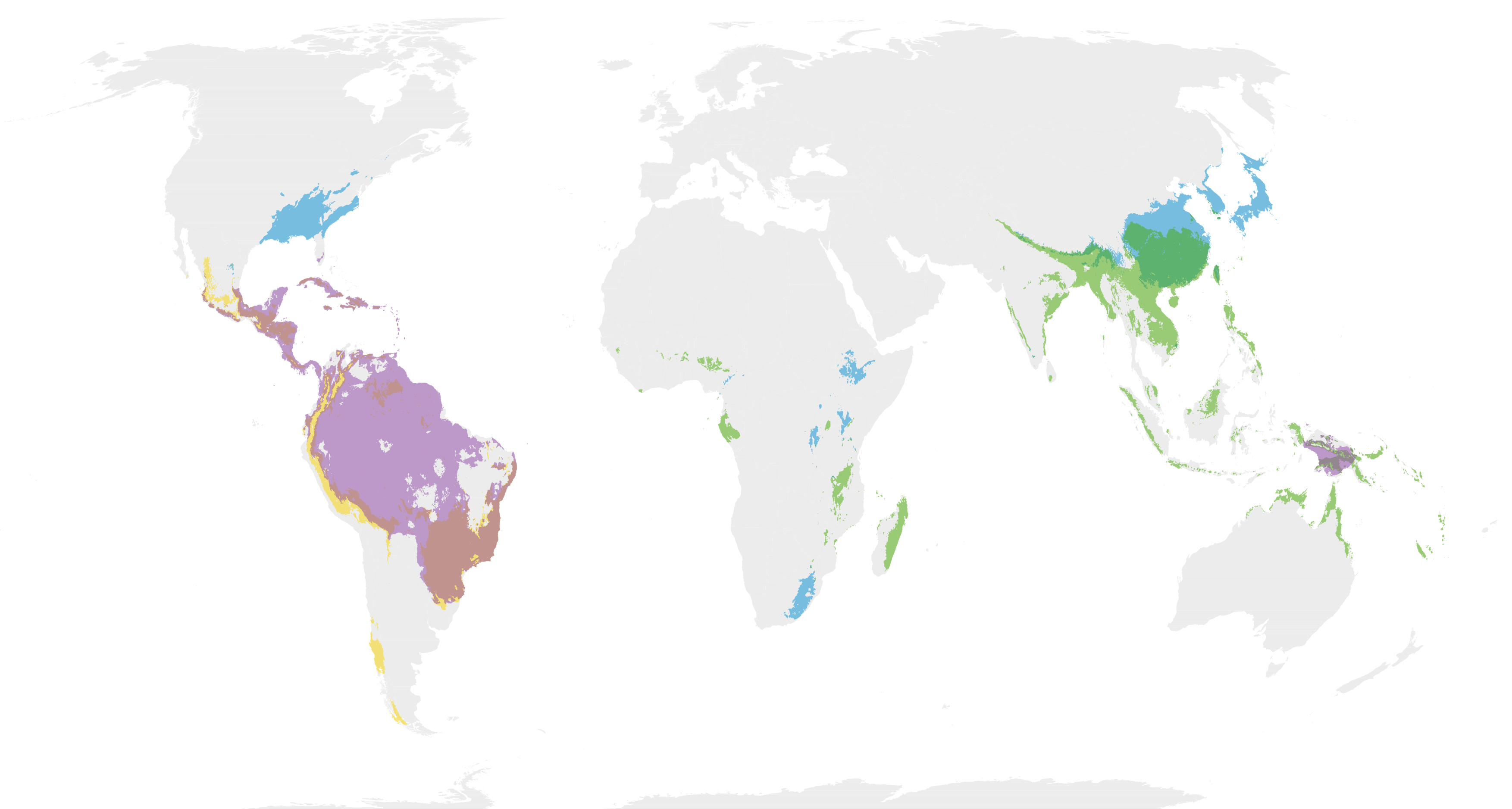

Supplement: Supplementary material 5 — Map images (png) of estimated Bambusoideae clade distributions [file bdj-13-e153436-s005.zip › Suppl. 4 - GIS Output Images/atlantic centered/areas/atlantic-all-areas.png]

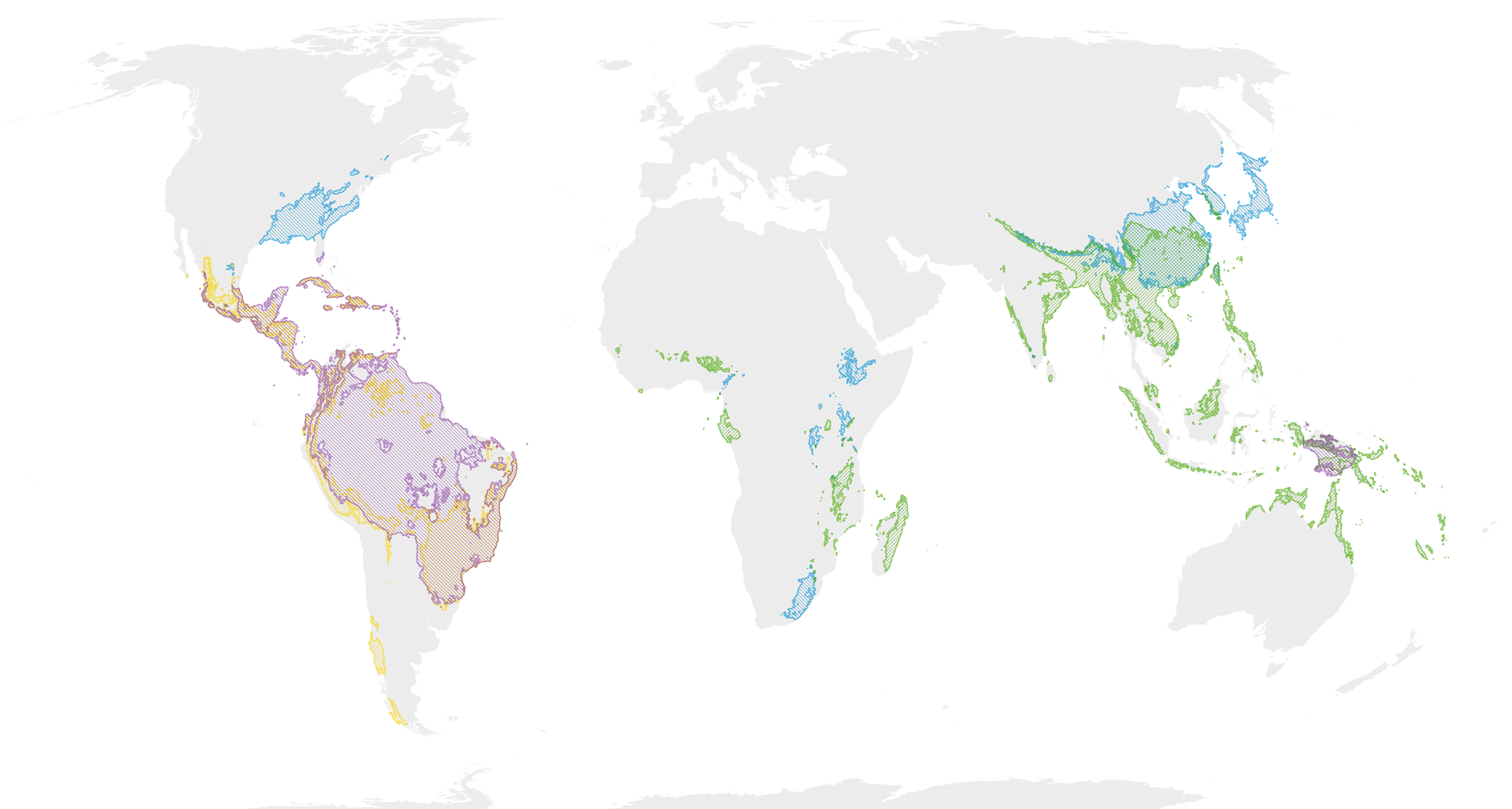

Supplement: Supplementary material 5 — Map images (png) of estimated Bambusoideae clade distributions [file bdj-13-e153436-s005.zip › Suppl. 4 - GIS Output Images/atlantic centered/areas/atlantic-all-crosshatched.png]

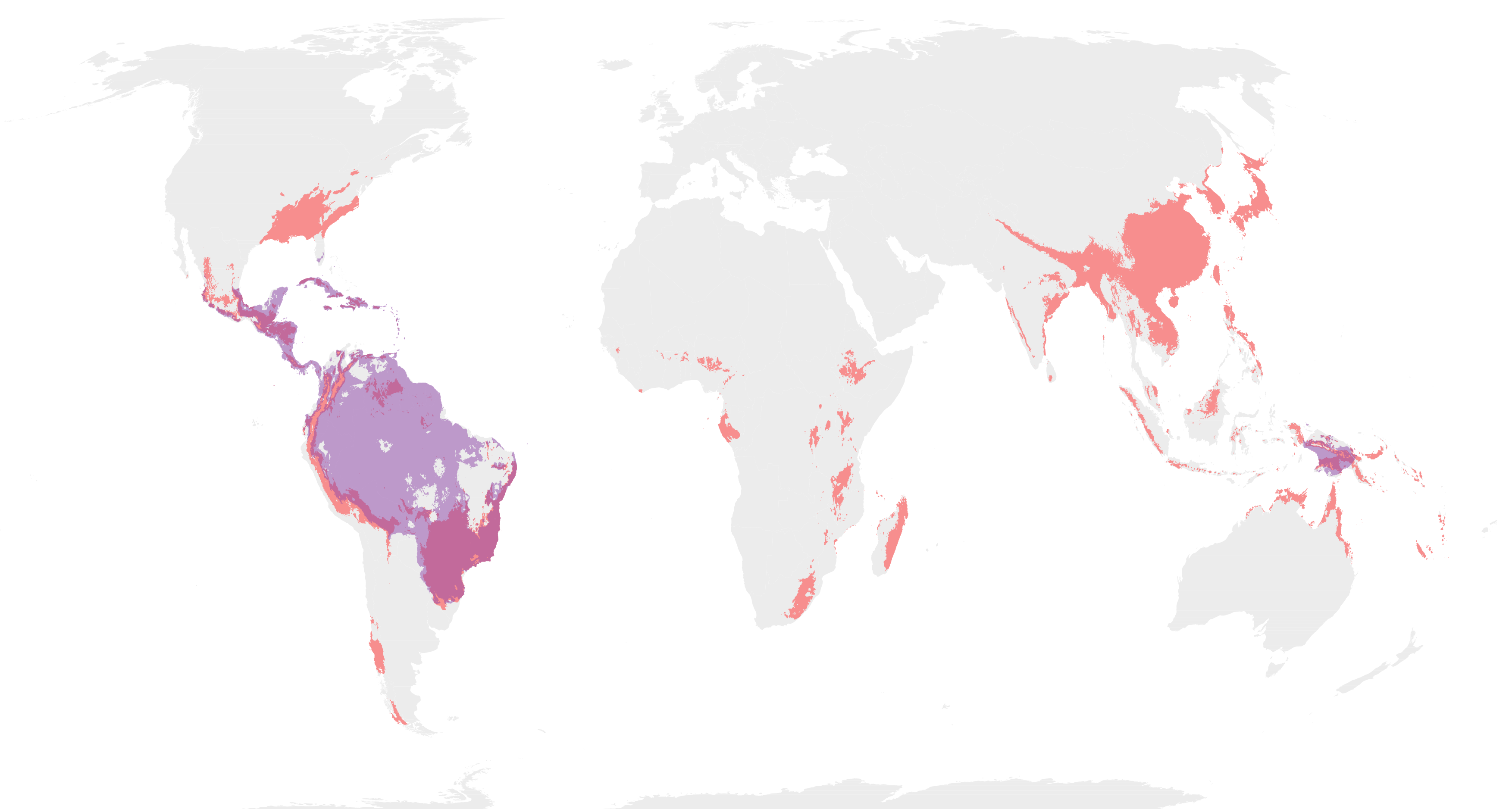

Supplement: Supplementary material 5 — Map images (png) of estimated Bambusoideae clade distributions [file bdj-13-e153436-s005.zip › Suppl. 4 - GIS Output Images/atlantic centered/areas/atlantic-woody_vs_herbaceous-areas.png]

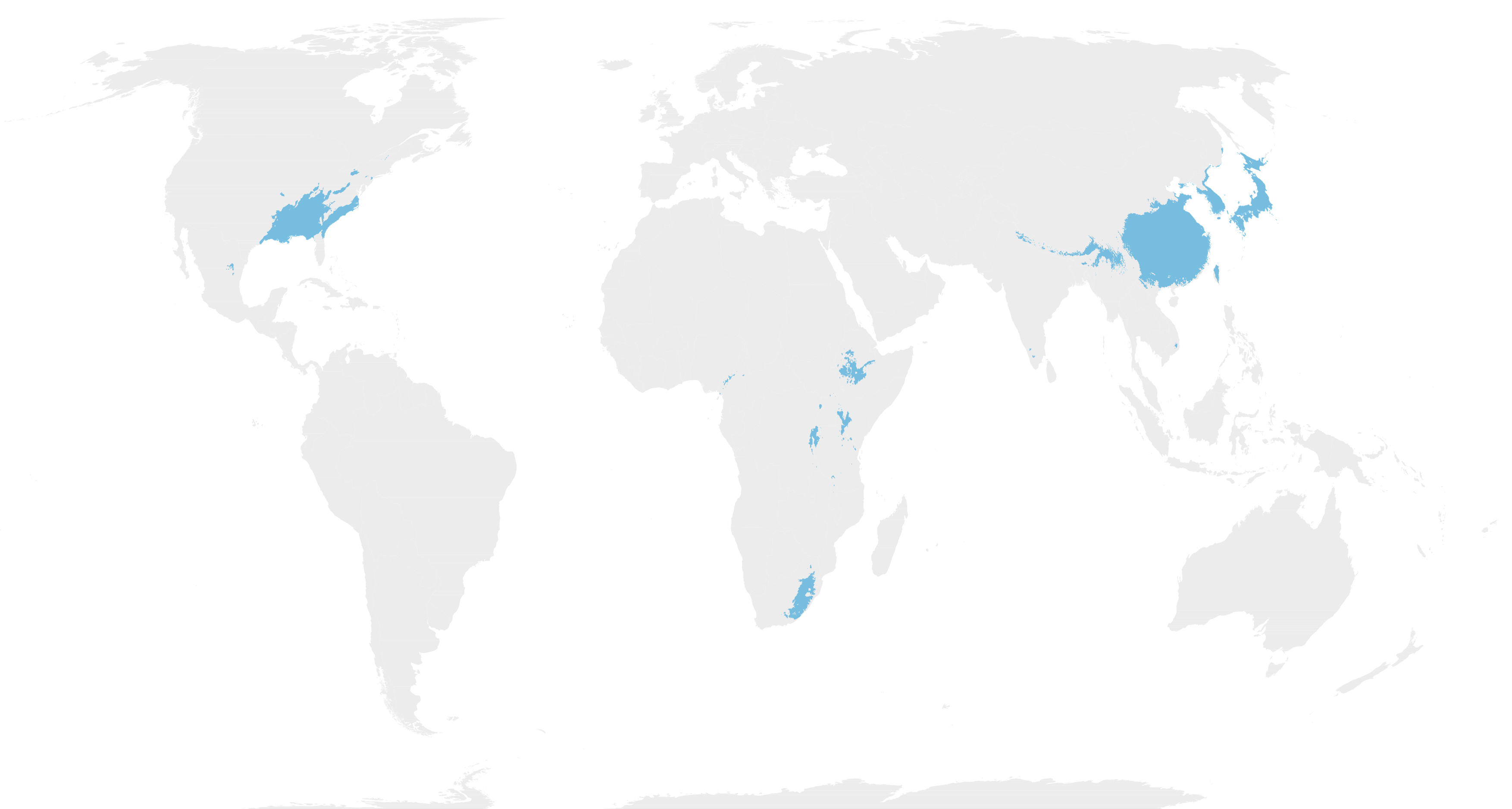

Supplement: Supplementary material 5 — Map images (png) of estimated Bambusoideae clade distributions [file bdj-13-e153436-s005.zip › Suppl. 4 - GIS Output Images/atlantic centered/areas/atlantic-twb-areas.png]

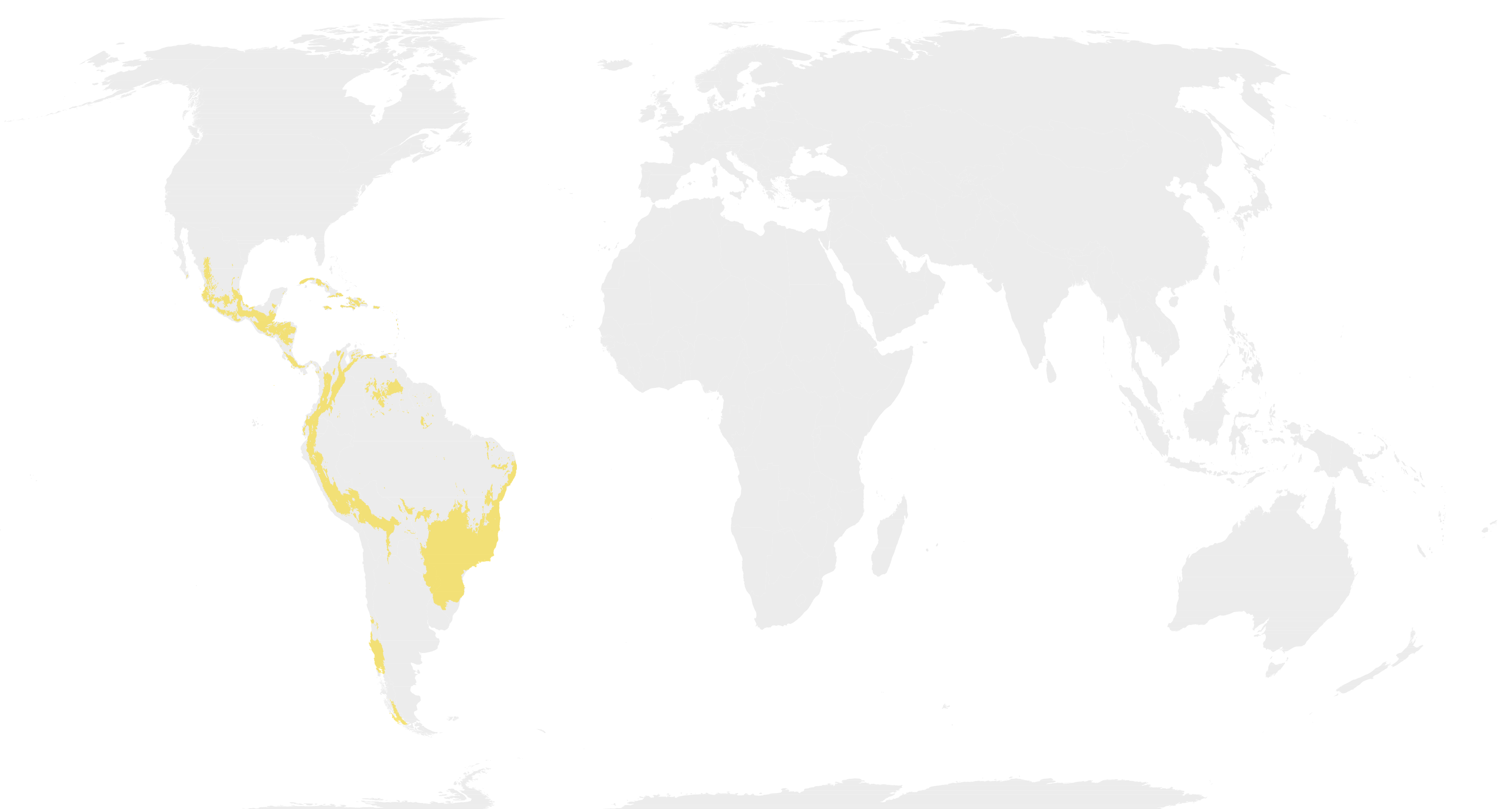

Supplement: Supplementary material 5 — Map images (png) of estimated Bambusoideae clade distributions [file bdj-13-e153436-s005.zip › Suppl. 4 - GIS Output Images/atlantic centered/areas/atlantic-nwb-areas.png]

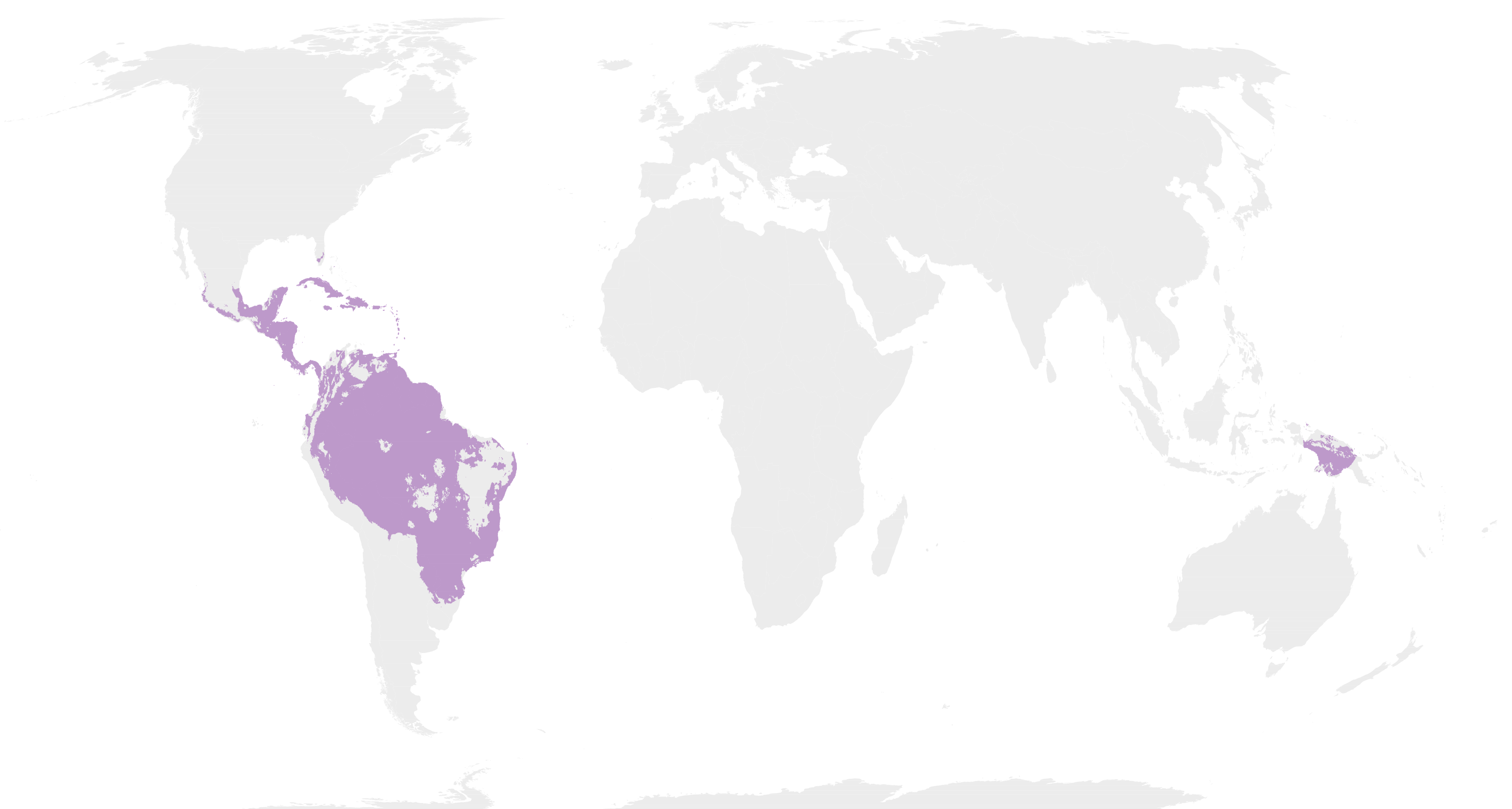

Supplement: Supplementary material 5 — Map images (png) of estimated Bambusoideae clade distributions [file bdj-13-e153436-s005.zip › Suppl. 4 - GIS Output Images/atlantic centered/areas/atlantic-herbaceous-areas.png]

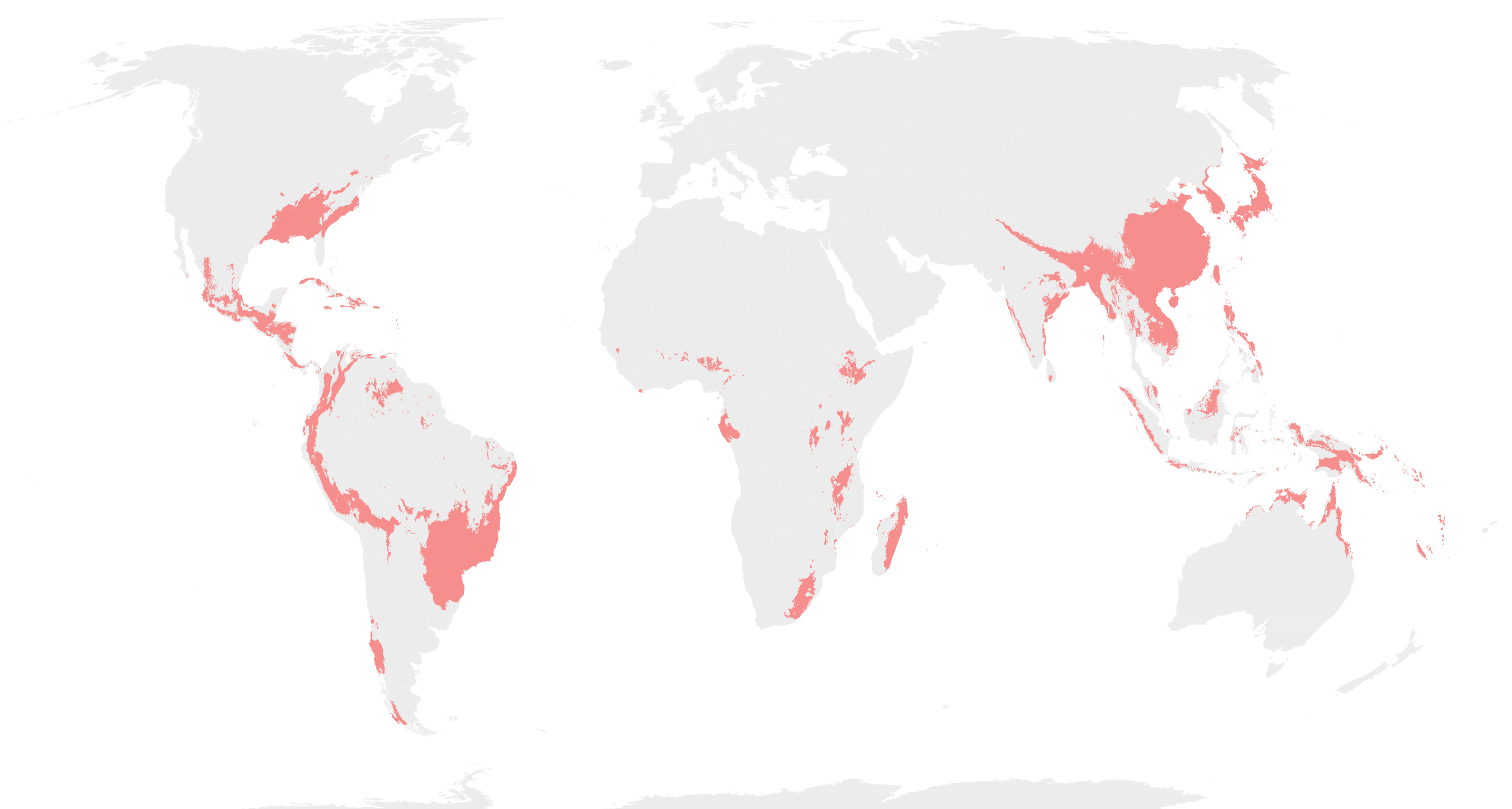

Supplement: Supplementary material 5 — Map images (png) of estimated Bambusoideae clade distributions [file bdj-13-e153436-s005.zip › Suppl. 4 - GIS Output Images/atlantic centered/areas/atlantic-woody-areas.png]

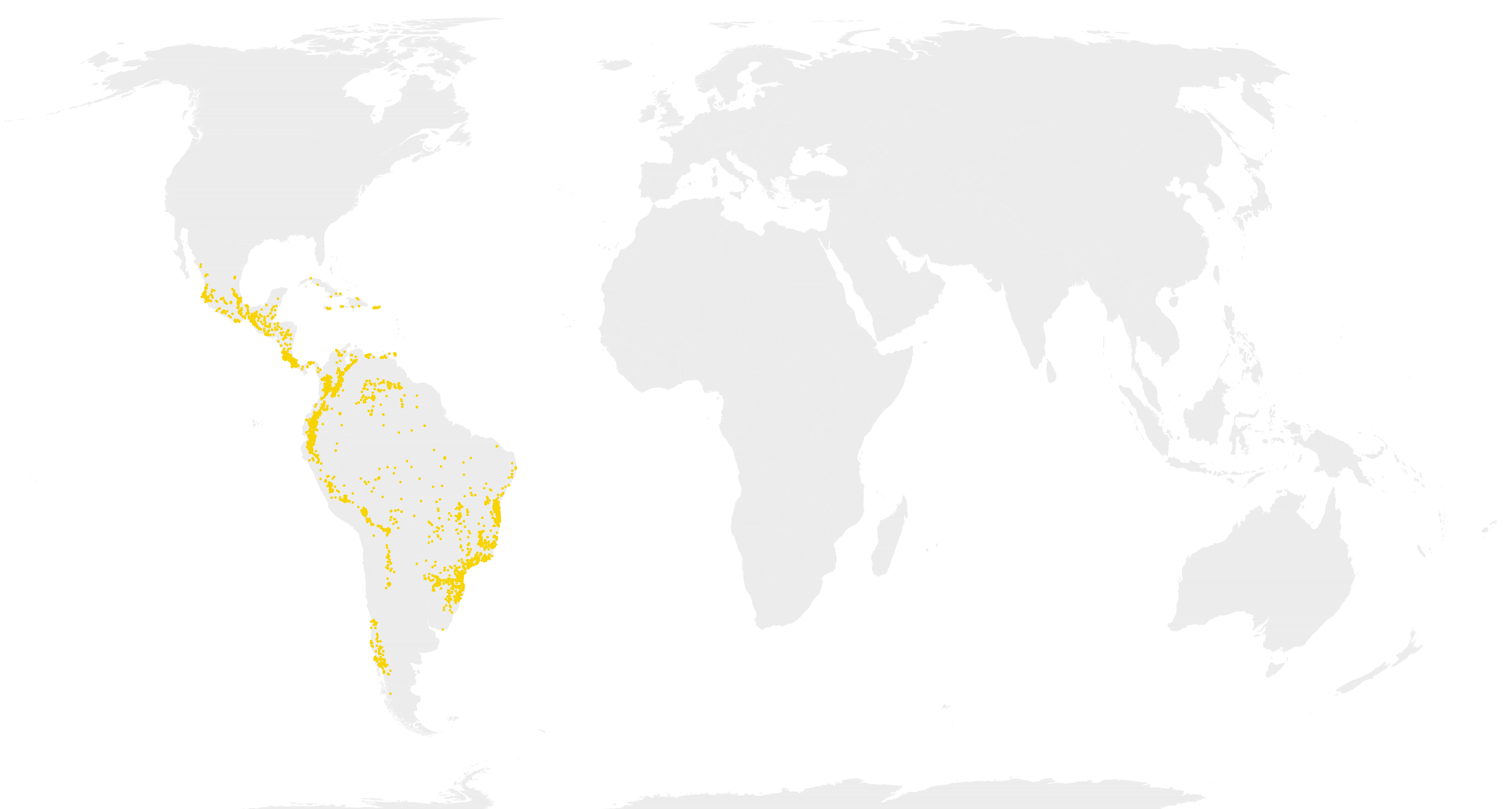

Supplement: Supplementary material 5 — Map images (png) of estimated Bambusoideae clade distributions [file bdj-13-e153436-s005.zip › Suppl. 4 - GIS Output Images/atlantic centered/points/atlantic-nwb-points.png]

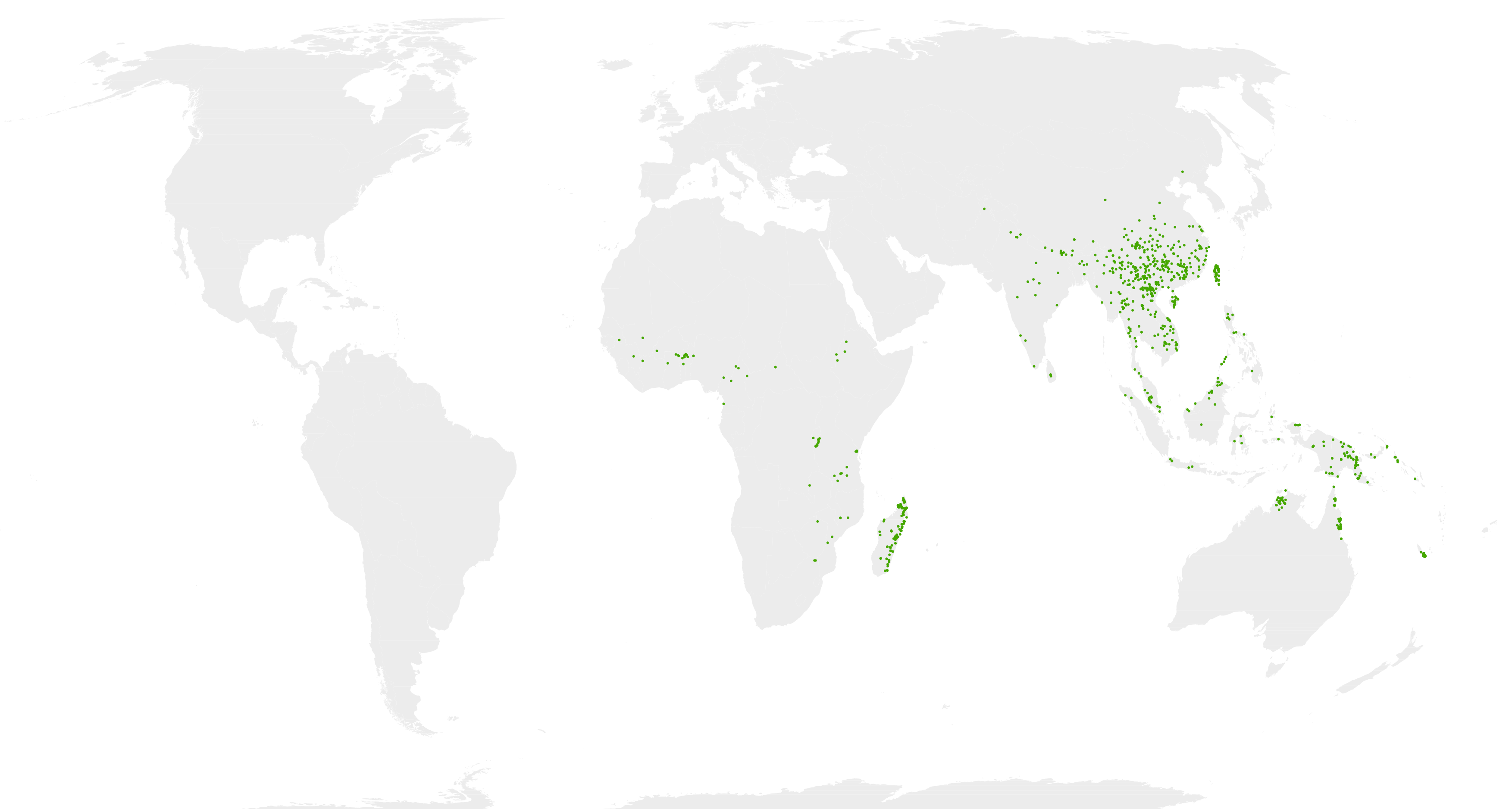

Supplement: Supplementary material 5 — Map images (png) of estimated Bambusoideae clade distributions [file bdj-13-e153436-s005.zip › Suppl. 4 - GIS Output Images/atlantic centered/points/atlantic-pwb-points.png]

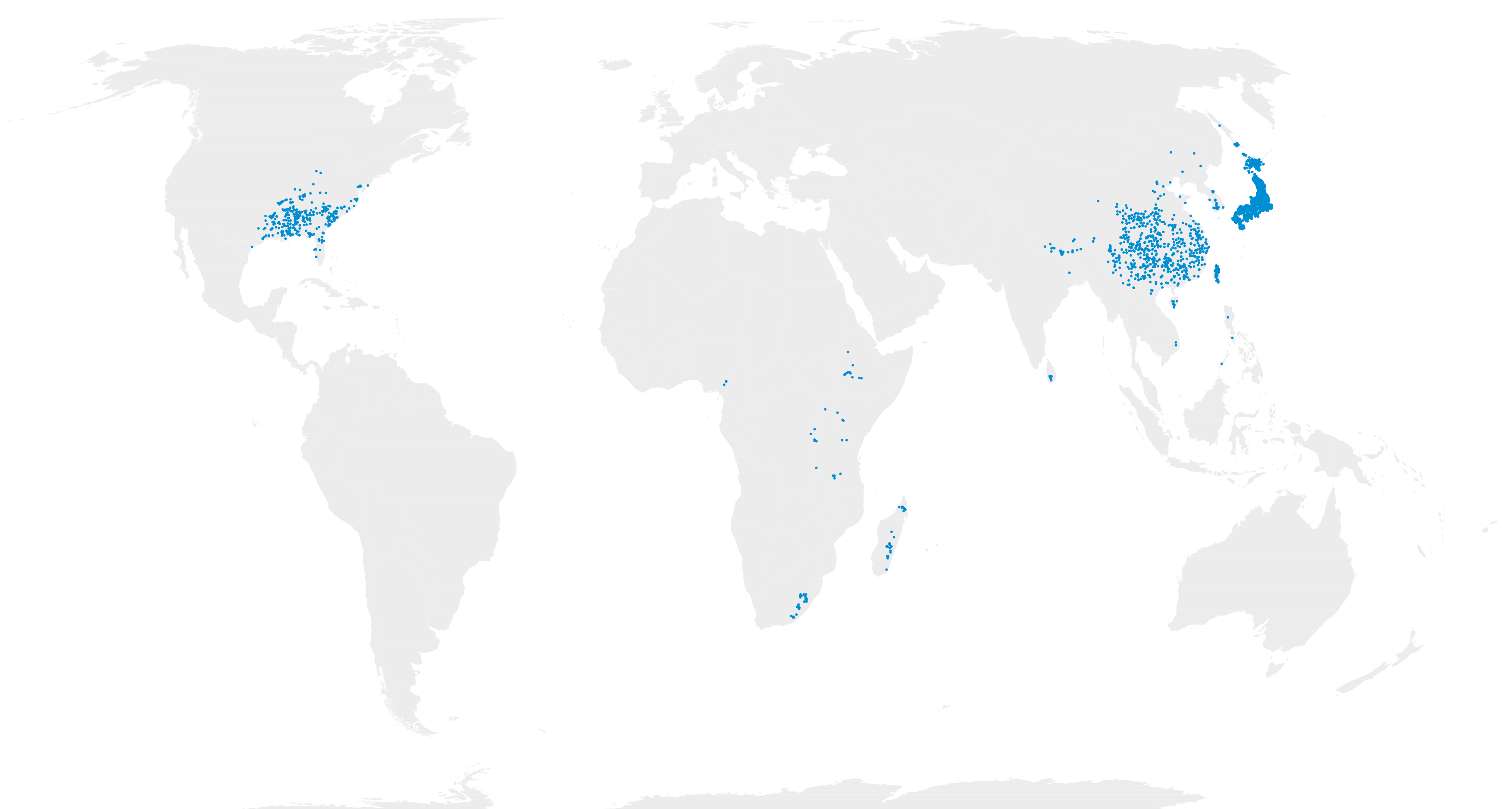

Supplement: Supplementary material 5 — Map images (png) of estimated Bambusoideae clade distributions [file bdj-13-e153436-s005.zip › Suppl. 4 - GIS Output Images/atlantic centered/points/atlantic-twb-points.png]

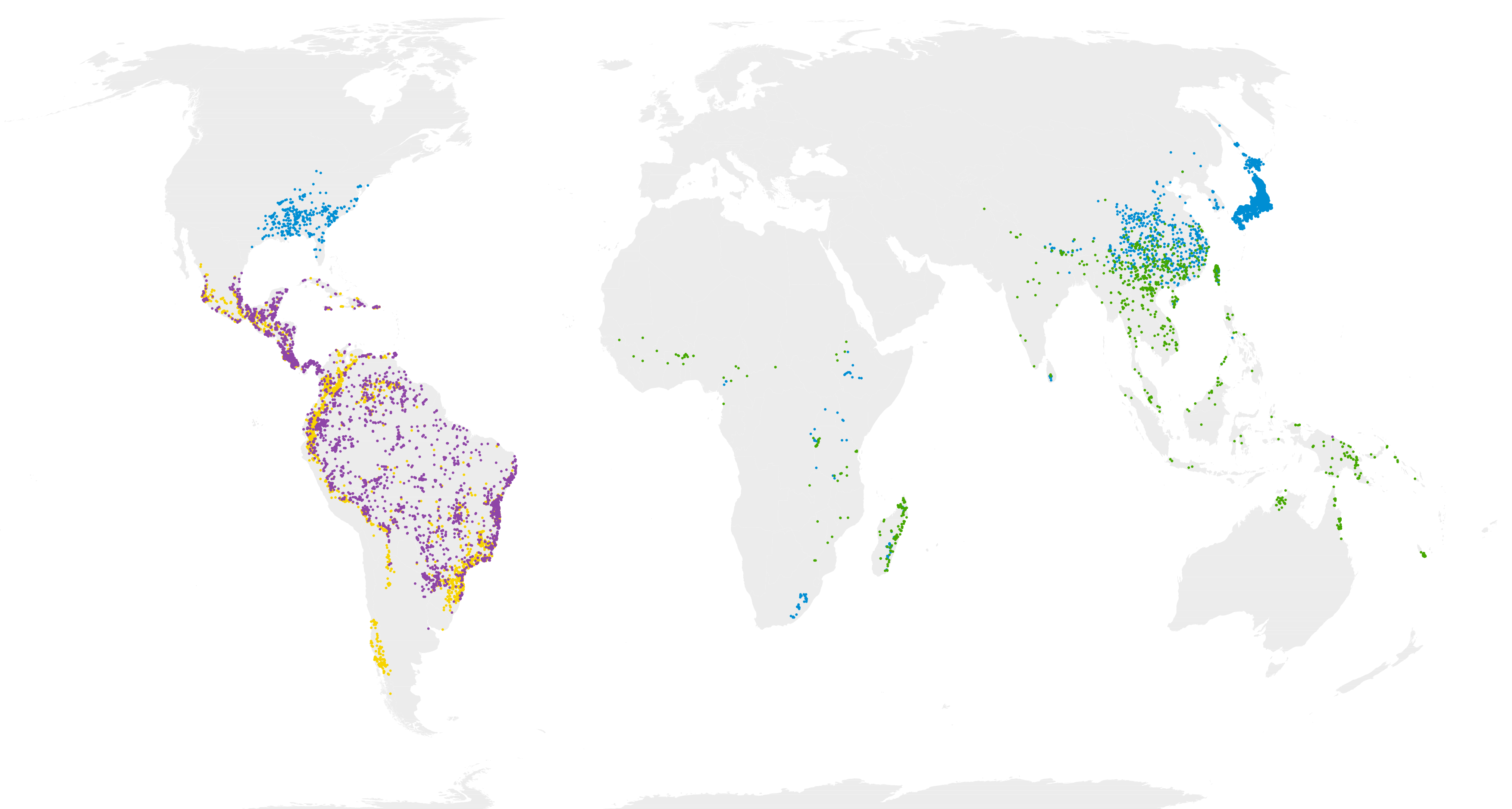

Supplement: Supplementary material 5 — Map images (png) of estimated Bambusoideae clade distributions [file bdj-13-e153436-s005.zip › Suppl. 4 - GIS Output Images/atlantic centered/points/atlantic-all-points.png]

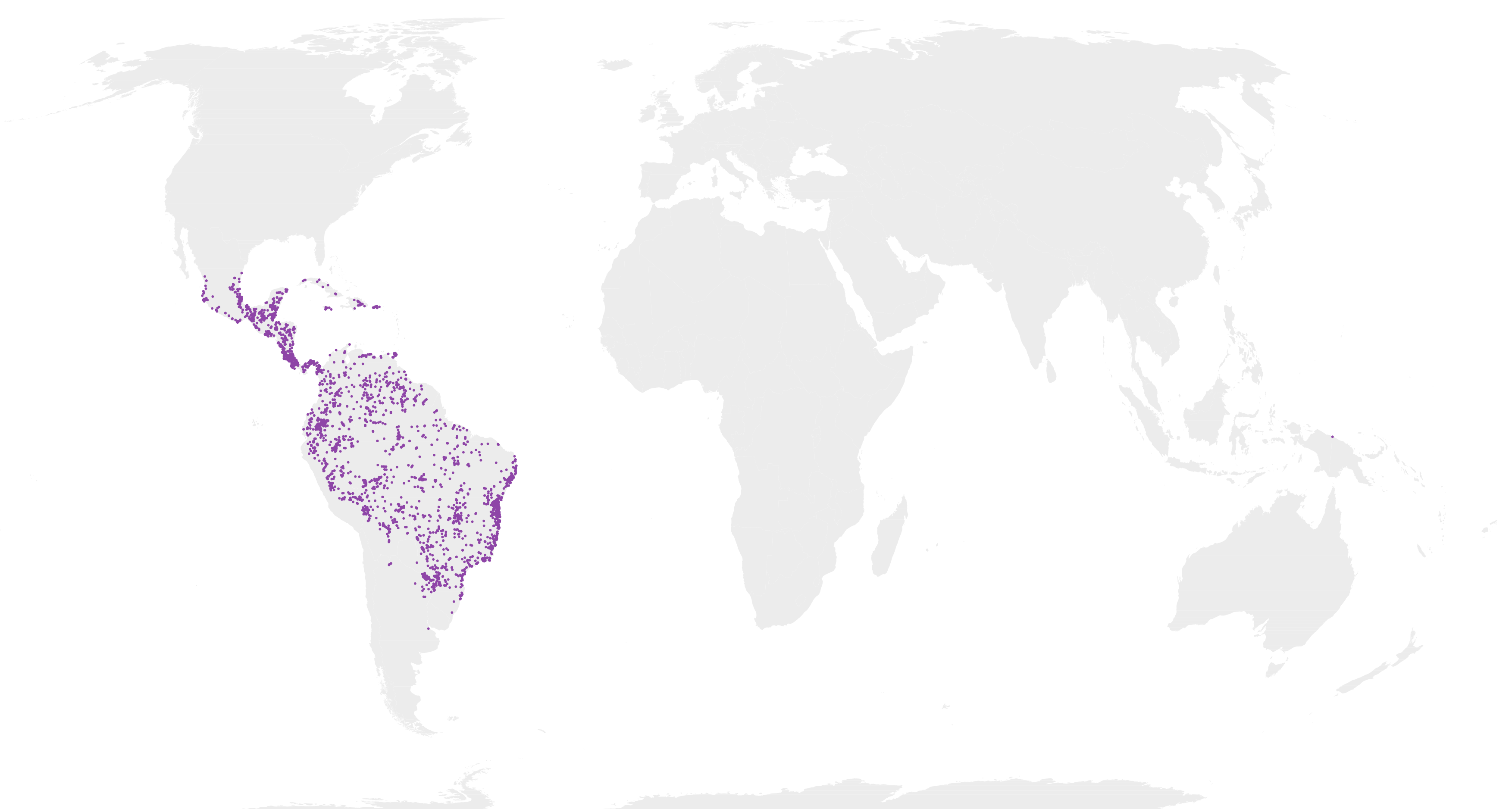

Supplement: Supplementary material 5 — Map images (png) of estimated Bambusoideae clade distributions [file bdj-13-e153436-s005.zip › Suppl. 4 - GIS Output Images/atlantic centered/points/atlantic-herbaceous-points.png]
